# Abstracts of the Cell Therapy Transplant Canada 2022 Annual Conference

**DOI:** 10.3390/curroncol29120783

**Published:** 2022-12-19

**Authors:** Stephanie A. Maier, Tobias Berg, Susan Berrigan, Jonathan Bramson, Chris Bredeson, Guy Cantin, Andrew Daly, Gwynivere A. Davies, Mahmoud Elsawy, Alejandro Garcia-Horton, Wilson Lam, Alix Lapworth, Kylie Lepic, Luciana Melo Garcia, Kirk R. Schultz, Ram Vasudevan Nampoothiri, Darrell White, Jean-Sébastien Delisle

**Affiliations:** 1 Cell Therapy Transplant Canada, Winnipeg, MB R3P 2R8, Canada; 2 Faculty of Health Sciences, McMaster University, Hamilton, ON L8S 4L8, Canada; 3 Hamilton Health Sciences, Hamilton, ON L8N 3Z5, Canada; 4 Alberta Precision Laboratories, Calgary, AB T2N 4Z6, Canada; 5 Ottawa Hospital Research Institute, Ottawa, ON K1H 8L6, Canada; 6 Centre Hospitalier Universitaire de Québec—Hôpital de l’Enfant-Jésus, Quebec City, QC G1J 1Z4, Canada; 7 Alberta Bone Marrow Transplant Program, Calgary, AB T2N 4N2, Canada; 8 Queen Elizabeth II Health Sciences Centre, Dalhousie University, Halifax, NS B3H 2Y9, Canada; 9 Princess Margaret Cancer Centre, Toronto, ON M5G 2C1, Canada; 10 MD Anderson Cancer Center, Houston, TX 77030, USA; 11 British Columbia Children’s Hospital Research Institute, Vancouver, BC V5Z 4H4, Canada; 12 Hôpital Maisonneuve-Rosemont, Université de Montréal, Montreal, QC H1T 2M4, Canada

**Keywords:** hematopoietic stem cell transplantation, cell therapy, GVHD, lymphoma, acute myeloid leukemia, myelodysplastic syndrome, acute lymphoblastic leukemia

## Abstract

On behalf of Cell Therapy Transplant Canada (CTTC), we are pleased to present the Abstracts of the CTTC 2022 Annual Conference. The conference was held in-person 15–18 June 2022, in Niagara Falls, Ontario. Poster authors presented their work during a lively and engaging welcome reception on Thursday, 16 June, and oral abstract authors were featured during the oral abstract session in the afternoon on Friday, 17 June 2022. Thirty-three (33) abstracts were selected for presentation as posters and six (6) as oral presentations. The top abstracts in each of four (4) categories, (1) Basic/Translational sciences, (2) Clinical Trials/Observations, (3) Laboratory/Quality, and (4) Pharmacy/Nursing/Other Transplant Support, received awards for both the oral and poster presentations. All of these were marked as “Award Recipient” with the relevant category. We congratulate all the presenters on their research and contribution to the field.

## Abstract 1 (Oral): CD56^bright^CD16^−^ Natural Killer Cells as an Important Regulatory Mechanism in Chronic Graft-Versus-Host Disease (Award Recipient—Basic, Translational)

**Madeline P. Lauener ^1^, Shima AzadPour ^1^, Sayeh Abdossamadi ^1^, Bernard Ng ^1^, Elena Ostroumov ^1^, Geoffrey D. E. Cuvelier ^2^, Megan K. Levings ^3,4,5^, Katherine N. MacDonald ^3,4,6^, Amina Kariminia^1^ and Kirk R. Schultz ^1^**

^1^ Michael Cuccione Childhood Cancer Research Program, British Columbia Children’s Hospital Research Institute, University of British Columbia, Vancouver, BC, Canada
^2^ CancerCare Manitoba, University of Manitoba, Winnipeg, MB, Canada
^3^ British Columbia Children’s Hospital Research Institute, University of British Columbia, Vancouver, BC, Canada
^4^ School of Biomedical Engineering, University of British Columbia, Vancouver, BC, Canada
^5^ Department of Surgery, University of British Columbia, Vancouver, BC, Canada
^6^ Michael Smith Laboratories, University of British Columbia, Vancouver, BC, Canada

**Background:** Chronic graft-versus-host-disease (cGvHD) is a major cause of morbidity and mortality after Hematopoietic Stem Cell Transplantation (HSCT). In 3 large human cohorts we identified increased numbers of a CD56^bright^, Granzyme B^−^, perforin^−^ NK cell population associated with the absence of cGvHD. We then identified the unique transcriptome of these NK cells using nanoString. This cell population is consistent with previously described regulatory NK cells (NK_regs_) and appears to be important in the induction of operational immune tolerance after HSCT.
**Purpose:** To effectively isolate and characterize the NK_reg_ cells according to phenotype and function to define the NK_reg_ population associated with a lack of cGvHD.
**Methods:** To determine cell surface markers for purely sorting CD56^bright^, Granzyme B^−^, perforin^−^ NK_reg_ cells, based on the previously determined NK_reg_ transcriptome, PBMC samples were stained for expression identification of the proteins CD56, CD16, CD3, perforin, Granzyme B, Granzyme K, GPR183R, CD127, CD62L, and CXCR3. To investigate the suppressive capacity of NK_reg_ cells against allogeneic CD4^+^ or CD8^+^ T cells, NK_reg_ cells (and the CD56^dim^ NK cell and T_reg_ cell controls) were isolated and co-cultured with CD3/CD28 activated CD4^+^ or CD8^+^ T cells. To determine if the suppression occurs through killing, the FITC Annexin V apoptosis detection kit was utilized. Additionally, the cytolytic ability of the NK cells was verified using a standard NK cell versus K562 cell killing assay. To determine if the suppression is contact dependent, the suppression assay procedures were followed using a 96-well transwell plate. Further, the soluble NKp44, NKp46, and GPR183 blockers were added to the suppression assay to determine the receptor dependence.
**Results:** NK_reg_ cells phenotypically associated with cGvHD suppression can be sorted with CD56 and CD16 cell surface antibodies (>95% purity). These NK_reg_ cells express Granzyme K, GPR183R, IL-7R, CXCR3, and CD62L with a lack of Granzyme B and perforin expression. The NK_reg_ cells engaged in a lack of cytotoxicity towards the CD4^+^ T cells and K562 cells, compared to the CD56^dim^ NK cells which lysed both targets. Further, the NK_reg_ cells suppressed CD4^+^ (but not CD8^+^) T cell proliferation comparable to T_reg_ cells through a cell-to-cell contact dependent mechanism, which was not reliant upon the NKp44, NKp46, or GPR183 receptors.
**Conclusions:** Our studies have phenotypically and functionally defined the NK_reg_ cell population associated with a lack of cGvHD and differentiated these cells from the classic, cytolytic NK cells. We have also shown the CD56^bright^CD16^−^ NK_reg_ cells to have a more selective, but comparable suppressive capacity to T_regs_, a cell subset that has been well studied in the context of GvHD and cell therapy. With further investigation we may decipher the mechanism of NK_reg_ suppression and optimize the expansion of NK_reg_ cells for use as a cell therapy for cGvHD.

**Figure 1:**
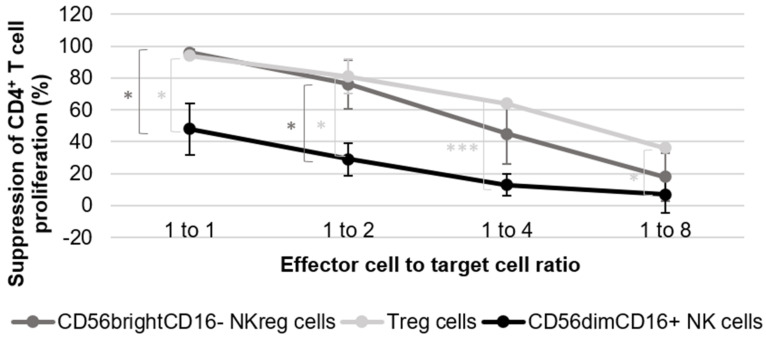
Evaluation of CD56^bright^CD16^−^ NK_reg_ cell ability to suppress CD4^+^ T cell proliferation. The graph is representative of the results of five experiments using cells derived from five healthy donors for the NK cell suppression assays, and two experiments using cells derived from two healthy donors for the T_reg_ cell suppression assay. The error bars are indicative of standard deviation, and the brackets are indicative of significant differences in suppression between the three different cell groups at all ratios tested (*p* < 0.05 = *, *p* < 0.0005 = ***). The graph directly compares the suppressive capacity of CD56^bright^CD16^−^ NK_reg_ cells, CD56^dim^CD16^+^ NK cells, and T_reg_ cells towards allogeneic CD4^+^ T cell proliferation at the 1:1, 1:2, 1:4, and 1:8 ratios as compared to the activated CD4^+^ T cells. The CD4^+^ T cell division index is scaled to 100% for calculation purposes, with all other condition indices scaled accordingly. This calculation was completed using the following standard formula: percentage of suppression = 1 − (division index of responder cells cultured with suppressor cells/division index of responder cells cultured without suppressor cells) × 100%.

**Figure 2:**
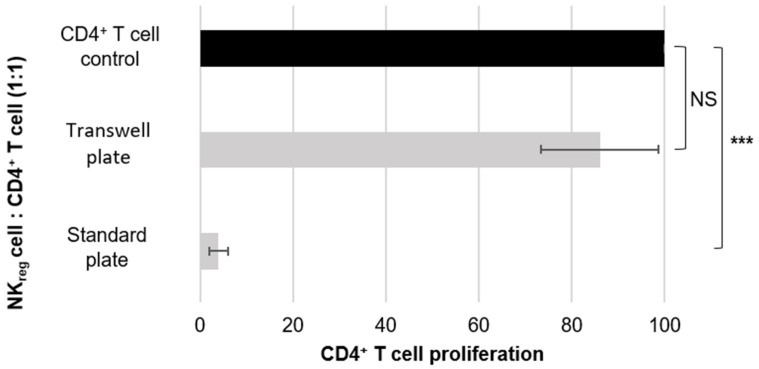
Evaluation of cell-to-cell contact dependence for CD56^bright^CD16^−^ NK_reg_ cell suppression. The graph is representative of the results of five different experiments using cells derived from five different donors. The error bars are indicative of standard deviation, and the brackets are indicative of significant differences in the suppressive capacity of NK_reg_ cells co-cultured with CD4^+^ T cells in a standard plate or a transwell plate as compared to the CD4^+^ T cell control (*p* < 0.0005 = ***, NS = not significant). The graph directly compares the suppressive capacity of the CD56^bright^CD16^−^ NK_reg_ cells towards allogeneic CD4^+^ T cells in a standard 96-well plate versus in a transwell 96-well plate.

## Abstract 2 (Oral): UM171 Expansion of Cord Blood Improves Donor Availability and HLA Matching for All Patients, including Minorities

**Maude Dumont-Lagacé ^1,2^, Albert Feghaly ^2^, Marie-Christine Meunier ^3^, Marcie Finney ^4^, Wouter Van’t Hof ^4^, Emeline Masson Frenet ^5^, Guy Sauvageau ^1,2,6,7^ and Sandra Cohen ^2,6,7^**

^1^ ExCellThera, Inc., Montreal, QC, Canada
^2^ Institute for Research in Immunology and Cancer (IRIC), Université de Montréal, Montreal, QC, Canada
^3^ Molecular Diagnostic Laboratory, Maisonneuve-Rosemont Hospital, Montreal, QC, Canada
^4^ Cleveland Cord Blood Center, Cleveland, OH, United States
^5^ National Cord Blood Program, New York Blood Center, New York, NY, United States
^6^ Department of Medicine, University of Montreal, Montreal, QC, Canada
^7^ Division of Hematology, Maisonneuve-Rosemont Hospital, Montreal, QC, Canada

**Background**: Cord blood (CB) stem cell transplantation offers a greater tolerance to HLA mismatches compared to adult-derived stem cell transplants (i.e., bone marrow or peripheral blood stem cells), with ≥4/6 or ≥5/8 HLA-matched CB transplants being regularly performed for patients without a matched donor. Unfortunately, most banked CB units contain a stem cell dose that is too small to treat adult patients safely. Ex vivo culture of CB stem cells with the small molecule UM171 is currently being developed to circumvent this cell dose issue.
**Purpose**: In this study, we retrospectively performed HLA matching simulations to assess how the minimal cell content requirements associated with UM171 CB expansion may improve usability of existing CB unit inventories and donor availability for patients of different races and ethnicities.
**Methods**: We analyzed a dataset of 58,971 adults for whom a donor search was initiated through the NMDP Be The Match registry against 142,941 CB units from major US public CB banks listed on the Be The Match registry. Matching was performed at the allele level for 8 HLA alleles (HLA-A, -B, -C and -DRB1).
**Results**: Our results show that by enabling selection of smaller CB units, UM171 expansion increases donor availability from 72% to 84% compared to single unmanipulated CB transplantation. UM171 expansion also increases donor availability compared to double CB transplantation, while enabling better HLA matching between donor and recipient. UM171 expanded CB appears particularly beneficial for racial and ethnic minority patients as CB availability increases from 53% to 78% for African Americans, from 66% to 85% for Hispanics and from 68% to 84% for Asians and Pacific Islanders, compared to single unmanipulated CB transplantation. UM171 expansion allows the use of small CB units, with as little as 1.5 × 10^7^ TNC/kg and 0.5 × 10^5^ CD34^+^ cells/kg. Thanks to a robust culture process, the lowest cell numbers that were successfully expanded for clinical use were 11.0 × 10^8^ TNC and 3.9 × 10^6^ CD34^+^ cells. UM171 expansion dramatically improves usability of CB units currently banked, as only 4.3% and 0.6% of banked CB units have sufficient cell dose for a 70 kg and 100 kg patient, respectively. UM171 raises this proportion to 53.8% and 20.2%, respectively, making CB banks potentially more cost-effective. Preliminary results indicate that expanded CB manufactured from CB units that would have been deemed to contain too little cells for single unmanipulated CB transplant performs as well as expanded CB manufactured from larger CB units, leading to similar time to neutrophil engraftment.
**Conclusions**: UM171 expansion allows the use of smaller CB units, thus giving clinicians more donor options to select from for their patient’s specific indication and clinical context, while also improving access to transplantation for racial and ethnic minorities.

## Abstract 3 (Poster): Clinical Impact of Adenovirus Infection in Pediatric Allogeneic Transplant Patients at a Single Institution

**Zahra Hudda, Sonata Jodele, Nathan Luebbering, Adam Lane, Stella Davies and Pooja Khandelwal**

Bone Marrow Transplantation & Immune Deficiency, Cincinnati Children’s Hospital Medical Center, Cincinnati, OH, United States

**Background:** Early adenovirus (AdV) reactivation post- allogeneic hematopoietic stem cell transplant (allo-HSCT) causes significant morbidity and mortality as cellular immunity is impaired due to incomplete immune reconstitution. AdV reactivation typically occurs in the gastrointestinal tract (GI) prior to detection in the blood, suggesting that pathogenesis involves localized tissue inflammation prior to systemic spread. We hypothesized that AdV reactivation triggers adverse events after allo-HSCT, such as acute GvHD and thrombotic microangiopathy (TMA), which are mediated by alterations in gut biomarkers such as interleukin-22 (IL-22), interleukin-22 binding protein (IL-22BP), and Reg3a. These biomarkers are important in regulating gut mucosal homeostasis, with variable pro or anti-inflammatory properties.
**Purpose:** Investigate the impact early adenovirus reactivation has on acute GvHD and TMA and identify an underlying mechanism.
**Methods:** We performed a retrospective analysis of 232 consecutive pediatric allo-HSCT patients transplanted between 2013–2018 at a single institution. AdV positivity was defined by measurable adenoviremia, detection on respiratory secretions and/or GI PCR at least once by day + 60. Secondary outcomes of OS, overall GvHD, GI GvHD and high-moderate TMA were evaluated using Cox regression analysis. Due to sample availability, subgroup analysis was performed on 108 consecutive patients by investigating their day + 30 plasma levels of IL-22, IL-22BP, and Reg3a via ELISA.
**Results:** Fifty-seven of the 232 (25%) of the patients were AdV+ with their patient demographics (Figure 1). The median day with range of adenovirus onset, GI GvHD and TMA were 19 (0–60), 24 (0–1856), and 34 (13–261) days, respectively. OS and aGvHD were not impacted by AdV reactivation with HR 1.21 (0.66–2.24), *p* = 0.539 and HR 1.14 (0.68–1.93), *p* = 0.620. However, acute GI GvHD and moderate-high degree of TMA were increased in the AdV+ population, HR 1.65 (1.03–2.66, *p* = 0.037) and HR 1.84 (1.09–3.10), *p* = 0.022, respectively. In the subset of patients who had biomarker evaluation at day + 30, IL-22 and Reg3a were elevated in the AdV+ cohort, (*p* = 0.021 and 0.004, respectively) and modestly positively correlated with each other (r = 0.17, *p* = 0.093). IL22-BP was significantly decreased in the AdV+ population, *p* = 0.012 (Figure 2).
**Conclusions:** Our analysis suggests that early AdV reactivation initiates a cascade of localized GI inflammation, which is shown by evidence of elevated IL-22 and Reg3a, and reciprocal reduction of IL-22BP, triggering a proinflammatory state (Figure 3). The GI inflammation likely increases the presence of inflammatory T cells and contributes to the increased risk for acute GI GvHD. GI inflammation leading to systemic AdV pathogenesis can plausibly contribute to a rise in cytokines and interferon levels driving TMA, as has been shown with BK virus. Our data offer a comprehensive explanation of the adverse impact early AdV reactivation.

**Figure 1:**
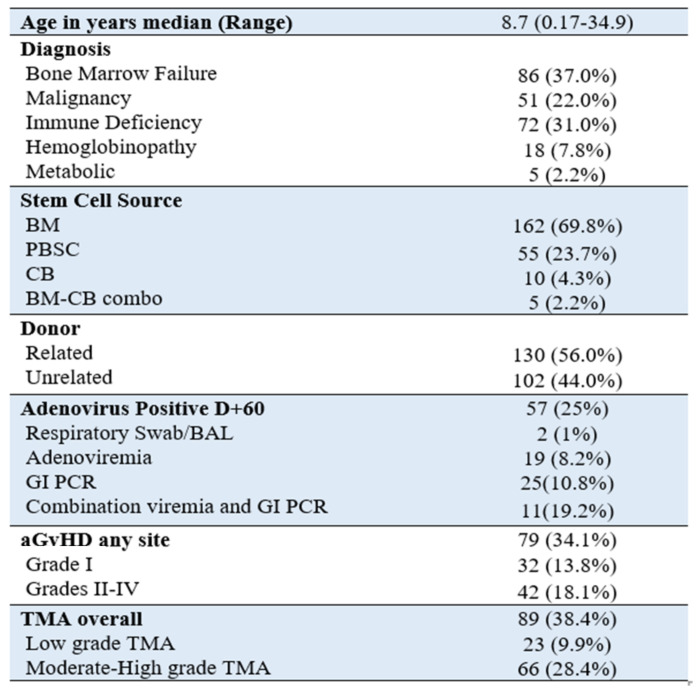
Patient demographics *n* = 232 patients.

**Figure 2:**
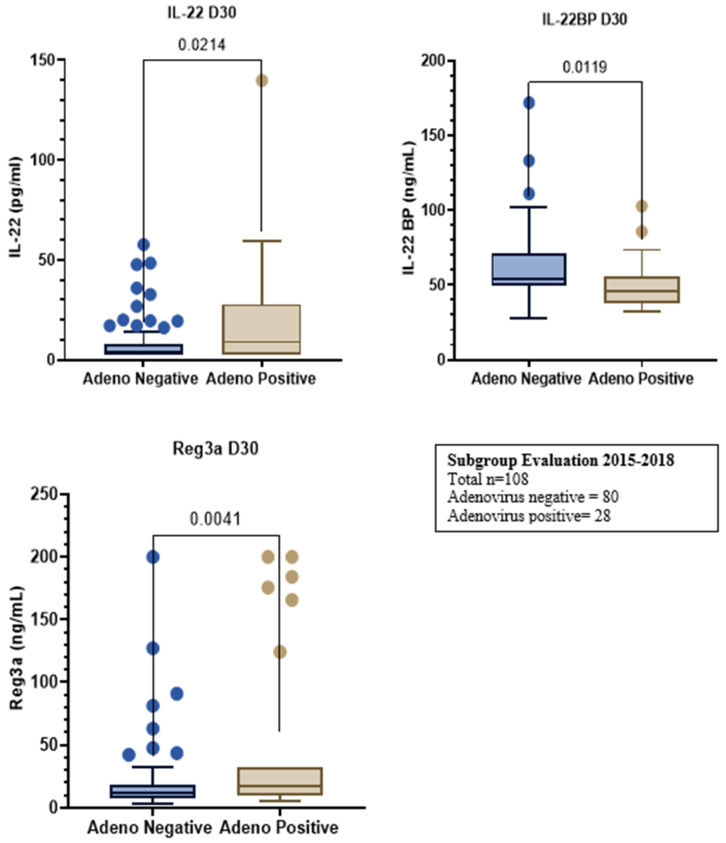
Subgroup evaluation from 2015–2018. Total n = 108; Adenovirus negative = 80, Adenovirus positive = 28.

**Figure 3:**
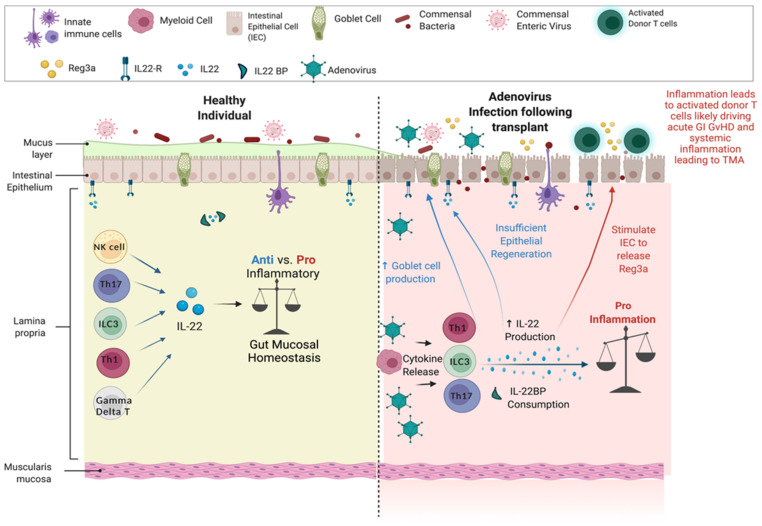
Heathy state versus transplant. Adenovirus infection following transplant produces elevated IL-22 and Reg3a, and reciprocal reduction of IL-22BP, triggering a proinflammatory state.

## Abstract 4 (Poster): Evaluating the Source of Soluble CD13 in Chronic Graft versus Host Disease

**Liam Johnston, Sayeh Abdossamadi, Vaishnavi Parthasarathy, Amina Kariminia, Bernard Ng, Elena Ostroumov, Madeline Lauener and Kirk R. Schultz**

British Columbia Children’s Hospital Research Institute, University of British Columbia, Vancouver, BC, Canada

**Background:** Chronic graft versus host disease (cGvHD) is a major cause of non-relapse mortality in hematopoietic stem cell transplantation (HSCT) patients. cGvHD has poor treatment options and is characterized by chronic inflammation resulting in sclerosis and multi-system organ damage. Previous adult and pediatric biomarker studies by our group and others have shown that an elevation in soluble CD13 (sCD13), a membrane-bound metalloproteinase, is linked to the development of cGvHD. We have shown that sCD13 maintains enzymatic activity, suggesting sCD13 activity in the plasma may impact donor anti-recipient immune responses, yet the cell source is not known. We hypothesized that a potential immune cell source for sCD13 would be found by identifying cell populations with altered CD13 expression when sCD13 is high.
**Purpose:** To ascertain the function of sCD13, as well as potential drug targets, we sought out to determine the identity of the source cells from which CD13 is cleaved in cGvHD.
**Methods**: We evaluated archived flow cytometry analyses and sCD13 enzymatic activity results obtained on pediatric patient samples from the Applied Biomarkers in Late Effects (ABLE/PBMTC 1202 trial cohort: *N* = total 240). Samples were placed into 6-month (*N* = 21) and onset of GvHD (*N* = 20) groups, as well as internal grouping of high vs. low sCD13 activity (≤0.64 mU/mL and ≥0.91 mU/mL, respectively, based on quartile calculation).
**Results:** When samples were compared between 6 months and onset, we found a decrease in the number of CD13^−^/CD56^+^ total NK cells (*p* = 0.0001) in the onset group compared to 6 months. Subpopulation analysis within the onset group did not reveal this relationship as being a potential source of CD13. This subpopulation analysis did, however, show a decrease in CD45^+^/CD66b^−^/CD14^−^/CD56^−^/CD3^−^/CD19^−^/CD13^+^ cells (*p* = 0.02), indicating hematopoietic cells as a potential source, and allowed us to exclude T cells, B cells, NK cells, macrophages, and neutrophils. In addition to the CD13^+^ population mentioned, we also found a population that presents with the same negative markers but is also CD13^−^ (CD45^+^/CD66b^−^/CD14^−^/CD56^−^/CD3^−^/CD19^−^/CD13^−^). This will require additional phenotyping to determine the cell type, and its potential role in cGvHD.
**Conclusions**: These results provide evidence for potential sources of sCD13 in cGvHD progression. We have determined the source is likely hematopoietic, excluding T cells, B cells, NK cells, macrophages, and neutrophils. Based on these results, we hypothesize that the most likely candidates are dendritic cells or basophils, however, a better understanding of the source cells of sCD13 will require further phenotypic evaluation.

## Abstract 5 (Poster): LSD1 Inhibition Enhances AML Immunogenicity for Maintenance after Allogeneic Stem Cell Transplantation

**Yu Yan ^1,2^, Kanwaldeep Singh ^1,2^, Maria Kleppe ^3^, Hugh Y. Rienhoff ^3^, Jonathan Bramson ^1,4,5^ and Tobias Berg ^1,2^**

^1^ Centre for Discovery in Cancer Research, McMaster University, Hamilton, ON, Canada
^2^ Department of Oncology, McMaster University, Hamilton, ON, Canada
^3^ Imago Biosciences, 329 Oyster Point Blvd, San Francisco, CA, United States
^4^ Department of Medicine, McMaster University, Hamilton, ON, Canada
^5^ McMaster Immunology Research Centre (MIRC), McMaster University, Hamilton, ON, Canada

**Background & Purpose:** Acute myeloid leukemia (AML) is an aggressive hematological malignancy with a dismal survival rate. Allogeneic hematopoietic stem cell transplantation (allo-HSCT) has become a cornerstone of curative AML treatment. In addition to reconstituting the hematopoietic system, allo-HSCT produces the desirable graft-versus-leukemia (GVL) effect, which is mainly mediated by alloreactive donor T cells. However, AML cells can often evade allogeneic immunosurveillance and lead to relapse of the primary disease. Recently, studies have demonstrated the impact of epigenetic modifications in driving AML immune evasion, which suggests the therapeutic roles of epigenetic inhibitors in post-transplant maintenance. Lysine-specific demethylase 1 (LSD1) is a promising therapeutic target for AML due to its role in regulating malignant cell differentiation and proliferation. We now aim to examine the ability of LSD1 inhibitors to promote T cell-mediated anti-leukemia immune responses.
**Method**: Using both human and murine AML cell lines, we now studied the effects of LSD1 inhibition on markers that are relevant to the current understanding of allogeneic immune evasion mechanisms. Leukemic cells were treated with increasing concentrations of the LSD1 inhibitor IMG-7289 (bomedemstat) and the respective markers were analyzed using quantitative PCR and/or flow cytometry.
**Results**: We found that pharmacological inhibition of LSD1 enhanced the expression of the master regulator of class II antigen presentation CIITA by 8-fold in OCI-AML3 and by 10-fold in HL-60. This led to an upregulation of HLA-DR in both cell lines as demonstrated by flow cytometry. At the same time, we observed upregulation of the costimulatory molecule CD86 in both human and murine cell lines. LSD1 inhibition also increased the production of the inflammatory cytokine IL-12 by 15-fold in MOLM-13 and by 6-fold in HL-60. Furthermore, we observed an upregulation of chemokine CXCL10 by 15-fold in MOLM-13 and 8-fold in HL-60. IL-12 and CXCL-10 are known to prime Th1 immune responses which are desired for promoting the GVL effect. We are currently investigating the functional significance of these results in ongoing experiments.
**Conclusions:** In conclusion, we have demonstrated that AML cells treated with LSD1 inhibitors acquire features of activated professional antigen-presenting cells which can enhance T cell activation. This makes LSD1 inhibition an interesting concept to pursue as a maintenance strategy after allo-HSCT.

## Abstract 6 (Poster): Biology of Immune Modulating Cells in Infants with and without Hypoxic Ischemic Encephalopathy

**Taylor Harris ^1,2^, Zhi-Juan Luo ^1^, Erika McCartney ^1,3^, Madison Denney ^1,2^, Amr El Shahed ^1^, Karin G. Hermans ^1^ and Donna A. Wall ^1,3^**

^1^ The Hospital for Sick Children, Toronto, ON, Canada
^2^ University of Waterloo, Waterloo, ON, Canada
^3^ Department of Immunology, University of Toronto, Toronto, ON, Canada

Hypoxic Ischemic Encephalopathy (HIE) is the leading cause of acquired neonatal brain injury. In this condition, the lack of oxygen to the brain around the time of birth results in a phase of primary energy failure marked by apoptosis and neuronal death. Following this initial phase, reperfusion activates the microglia and a potent inflammatory cascade. This secondary energy failure due to inflammation is responsible for the majority of neuronal cell death and is, therefore, a target for intervention. Immature myeloid cells that expand in inflammatory conditions can exert a profound anti-inflammatory response and may be harnessed to minimize the second phase of neuronal cell death.
This pilot study measured circulating hematopoietic stem/progenitor cells (HSPC) and innate immune regulatory cells (G-MDSCs, M-MDSCs, Tregs) in the first three days of life by flow cytometry to establish the number of circulating HSPCs and immunosuppressive cells. An initial analysis of 60 newborn infants treated for a range of disorders demonstrated a decline in circulating CD34^+^ cells with age, ranging from 1–2% in cord blood (birth sample) to 0.05% by 10 h of life as HSPCs home to the bone marrow. Granulocytic myeloid-derived suppressor cells (G-MDSCs) (CD15^+^ HLADR^−^, LOX-1^+^) follow a less pronounced decline over time averaging 1871 G-MDSCs/µl of blood in the first 24 h of life compared to 866 G-MDSCs/µL of blood by three days of age. Interestingly, this population appears to be larger in infants with HIE (red points) compared to non-HIE infants. In contrast, both monocytic myeloid-derived suppressor cells (M-MDSCs) (CD14^+^, HLADR^−^) and T-regulatory cells (Tregs) (CD25^+^, CD127^−^) circulate in lower ranges and remain stable over time consisting of 3.47–1079 MDSCs/µL and 8.34–234 Tregs/µL. This data establishes a baseline of circulating HSPCs and immunosuppressive populations in the first three days of life. In the next phase of this project, samples will be collected and analyzed exclusively from infants with severe HIE over several time points to establish ranges in this patient population compared to controls. Overall, this data provides preliminary information for a prospective clinical trial with the objective of harnessing host immune modulating cells for potential use as immunotherapy for infants with HIE.

## Abstract 7 (Poster): All CD34^+^ Cells Are Not Equal in Hematopoietic Stem Cell Grafts (Award Recipient—Basic, Translational)

**Marie Rachel ^1,2^, Erika McCartney ^1,2^, Raymond Kung ^1^, Donna A. Wall ^1,2^ and Karin G. Hermans ^1^**

^1^ The Hospital for Sick Children, Toronto, ON, Canada
^2^ Department of Immunology, University of Toronto, Toronto, ON, Canada

**Introduction:** Hematopoietic stem cell transplant (HSCT) is a life-saving procedure used to restore hematopoiesis in patients having undergone high-dose myeloablative chemotherapy. Current standard of care dosing of hematopoietic progenitors is based on the count of CD34^+^ cells/kg, in general administering a minimum of 2.5 million CD34^+^ cells/kg for growth factor-mobilized products. However, CD34 is expressed on a range of hematopoietic stem/progenitor cells (HSPC) which have variable stemness and repopulation capacity. The objective of this study is to assess the quality and quantity of CD34^+^ cell populations present in autologous versus healthy allogeneic hematopoietic stem cell sources to further understand the determinants of HSCT outcomes, with the hope of improving engraftment and hematopoietic recovery post-transplant.
**Methods:** CD34^+^ HSPC from previously cryopreserved G-CSF mobilized autologous and allogeneic HSPC products and cord blood were positively selected for CD34^+^ cells and then analyzed by flow cytometry using an 8-antibody CD34 hierarchy panel to characterize early hematopoietic progenitor populations within the grafts.
**Results:** Analysis of grafts prior to CD34 isolation revealed significant differences in CD34^+^ content of the pediatric autologous, allogeneic, and cord blood stem cell products. The proportion of CD34^+^ cells in autologous grafts (mean 20.8%; range 5–41.3%) was significantly higher than in cord blood (0.7%; 0.15–1.3%) and grafts collected from healthy donors after G-CSF mobilization (2.3%; 0.8–3%). Next, we analyzed the hierarchy of the CD34^+^ selected cells. Long-term hematopoietic stem cells (LT-HSC, defined as CD34^+^CD38^−^CD90^+^CD45RA^−^CD49f^+^) repopulate the bone marrow and initiate long-term hematopoietic reconstitution. This subset was a minor subpopulation of all CD34^+^ cells but varied between the grafts. The proportion of LT-HSC was highest in cord blood (1.5% of CD34^+^ cells; 1.02–2.14%) compared to allogeneic healthy donors (0.8%, 0.3–1.3%) and autologous donors (0.6%, 0.06–0.9%). Of note, the autologous transplant donors were collected on count recovery following both chemotherapy and G-CSF mobilization. Additionally, we observe significant differences in other CD34^+^ populations, including granulocyte/monocyte and megakaryocyte/erythroid progenitors.
**Conclusions:** CD34^+^ graft content varies depending on HSC source, with autologous mobilized products containing a much higher proportion of CD34^+^ cells. Grafts with high CD34 content have fewer non-hematopoietic cells in the treatment dose which can potentially impact immunologic recovery post-HSCT. In addition, analysis of the CD34^+^ hematopoietic hierarchy shows that most CD34^+^ cells are committed progenitor cells with limited repopulation potential and myeloid skewing. Moreover, pediatric autologous grafts have the lowest content LT-HSC which provide life-long hematopoiesis.

**Figure 1:**
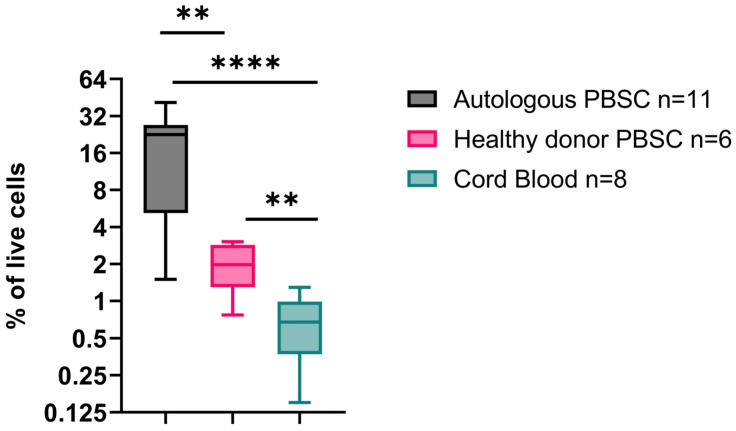
CD34^+^ content in autologous, healthy donor allogeneic and cord blood stem cell products. *p* < 0.05 = **, *p* < 0.0005 = ****.

## Abstract 8 (Poster): The EBMT Disease Risk Stratification System (DRSS) Allows Prediction of Relapse after Allogeneic Hematopoietic Cell Transplantation for Acute Myeloid Leukemia and Myelodysplastic Syndrome

**Yasmine Kadri, Michelle Phan, Julie Bergeron, Nadia Bambace, Léa Bernard, Sandra Cohen, Jean-Sébastien Delisle, Thomas Kiss, Sylvie Lachance, Denis-Claude Roy, Jean Roy, Guy Sauvageau, Olivier Veilleux and Imran Ahmad**

Institut Universitaire d’Hémato-Oncologie et de Thérapie Cellulaire, Hôpital Maisonneuve-Rosemont, Université de Montréal, Montreal, QC, Canada

**Background:** Using EBMT registry data, the DRSS has been proposed to predict relapse risk after allogeneic hematopoietic cell transplantation (HCT) across disease subtypes and remission states ordered in 55 categories and 5 risk levels [1]. For acute myeloid leukemia (AML) the DRSS combines ELN risk group, remission rank, and de novo vs. secondary AML in 19 categories and for myelodysplastic syndrome (MDS) it includes 5 categories.
**Purpose:** We sought to determine the reproducibility of the DRSS in a cohort of subjects transplanted for AML or MDS.
**Methods:** Data from a single-center cohort of consecutive adult AML & MDS patients transplanted between 1 July 2015 and 30 June 2020 was analyzed retrospectively. Baseline characteristics and outcomes were extracted, and Fine-Gray regression was used to determine the association between cumulative incidence of relapse (CIR) and patient, disease, and transplant characteristics. Model selection techniques were used to select the least number of significant predictors of CIR.
**Results:** In this cohort of 134 patients, median follow-up was 2.7 and CIR was 26 % at 4 years (95% confidence interval: 18–35). DRSS was independently associated with CIR after adjustment for several covariates: patient age > 60 years, donor type, regimen intensity, secondary disease, and graft source. DRSS was divided into 3 groups with CIR at 4 years of 18% (95%CI: 8–30) for low risk, 32% (CI: 19–46) for intermediate 1 and 2 risks, and 40% (CI: 17–63) for high and very high-risk groups. Univariate graphic representation of CIR according to DRSS is shown in Figure 1.
**Conclusions:** In adults with AML and MDS, cumulative incidence of relapse after allogeneic HCT can be predicted by DRSS across all donor types and age groups. DRSS is a useful tool for the assessment of disease relapse risk in clinical studies, validated for AML and MDS patients.

**Figure 1:**
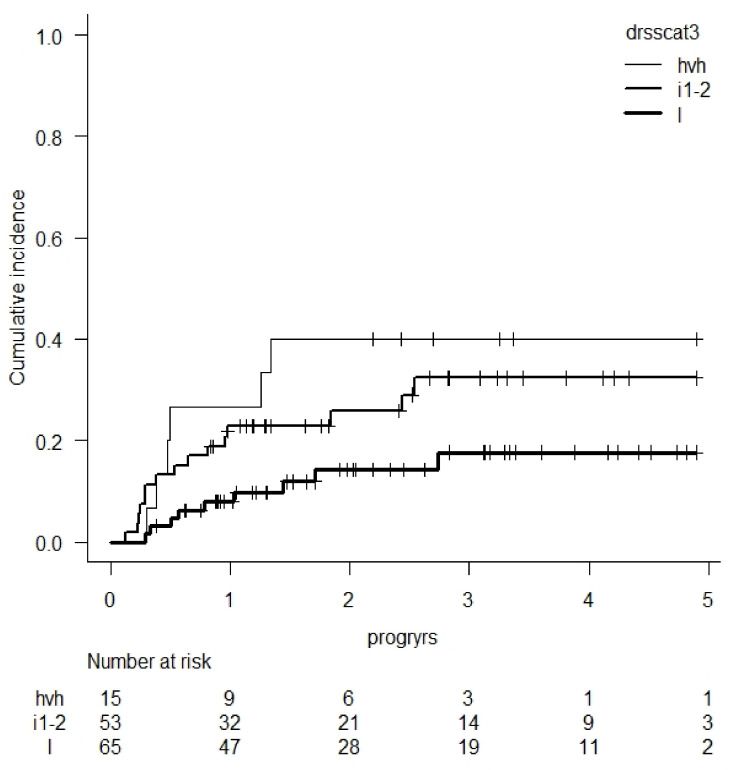
CIR according to DRSS score (l = low, i1-2 = intermediate 1 & 2, hvh = high & very high).

**References:**
  1. Shouval, R.; Fein, J.A.; Labopin, M.; Cho, C.; Bazarbachi, A.; Baron, F.; Bug, G.; Ciceri, F.; Corbacioglu, S.; Galimard, J.E.; et al. Development and validation of a disease risk stratification system for patients with haematological malignancies: a retrospective cohort study of the European Society for Blood and Marrow Transplantation registry. *Lancet Haematol.* **2021**, *8*, e205–e215. Erratum in: *Lancet Haematol.* **2021**, *8*, e393. https://doi.org/10.1016/S2352-3026(20)30394-X.

## Abstract 9 (Poster): Higher Donor Age with a Cut-Off of 50 Years Is Associated with Increased Non-Relapse Mortality after Allogeneic Hematopoietic Cell Transplantation for Acute Myeloid Leukemia and Myelodysplastic Syndrome

**Yasmine Kadri, Michelle Phan, Julie Bergeron, Nadia Bambace, Léa Bernard, Sandra Cohen, Jean-Sébastien Delisle, Thomas Kiss, Sylvie Lachance, Denis-Claude Roy, Jean Roy, Guy Sauvageau, Olivier Veilleux and Imran Ahmad**

Institut Universitaire d’Hémato-Oncologie et de Thérapie Cellulaire, Hôpital Maisonneuve-Rosemont, Université de Montréal, Montreal, QC, Canada

**Background:** The association between donor age (DA) and allogeneic hematopoietic cell transplantation (HCT) outcomes is controversial.
**Purpose:** We sought to determine the association between DA and the incidence of non-relapse mortality (NRM) in adult acute myeloid leukemia (AML) and myelodysplastic syndrome (MDS) patients treated with HCT
**Methods:** Data from a single-center cohort of adult AML and MDS patients consecutively transplanted (1st HCT) between 1 July 2015 and 30 June 2020 was analyzed retrospectively. Baseline characteristics[ia2] and outcomes were extracted, and cumulative incidence CI of NRM (CI-NRM) was estimated using death before relapse as competing risk. Fine-Gray multivariate models and model selection techniques were used to determine the association between DA and cumulative incidences (CIR) of NRM and GVHD. Confounding variables included were age-adjusted HCT comorbidity index (aaHCT-CI), Karnofsky performance score (KPS), donor type, conditioning intensity, cytomegalovirus (CMV) status in recipient and gender mismatch.
**Results:** Pre-HCT patient characteristics (*n* = 134) are presented. Median follow-up was 2.7 years, CI-NRM was 18% at 5 years from HCT (95% confidence interval (CI): 10–29) and survival at 5 years was 61% (CI: 48–72). DA was independently associated with CI-NRM (hazard ratio (HR) at 50 years 3.43, *p* = 0.01; at 60 years 3.98, *p* = 0.02). The HR for NRM increased with DA reaching statistical significance at 50 years (Figure 1a). The only other statistically significant variable independently associated with NRM was aaHCT-CI (HR 1.90, *p* = 0.04). No association was found between DA and incidences of graft-versus-host disease (GVHD).
**Conclusions:** A donor ≥ 50 years of age for patients transplanted for AML or MDS increases the risk of NRM. The pathological mechanisms responsible for this association remain to be elucidated and do not seem to be mediated by GVH.

**Figure 1:**
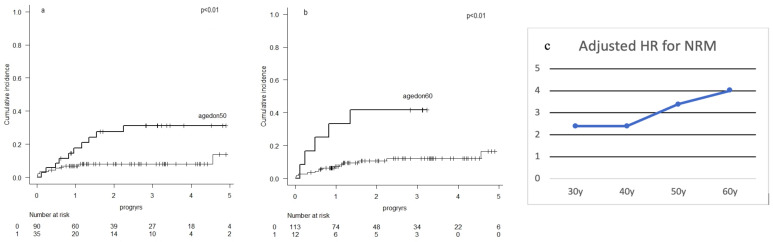
Cumulative incidence of non-relapse mortality with donors aged <50 years vs. ≥50 years (**a**) <60 years vs. ≥60 years (**b**). Adjusted hazard ratios (HR) of various donor ages for the association with non-relapse mortality (NRM) (**c**).

## Abstract 10 (Poster): Primary Analysis of ZUMA-7: A Phase 3 Randomized Trial of Axicabtagene Ciloleucel (Axi-Cel) versus Standard-of-Care Therapy in Patients with Relapsed/Refractory Large B-Cell Lymphoma

**Frederick L. Locke ^1^, David B. Miklos ^2^, Caron Jacobson ^3^, Miguel-Angel Perales ^4^, Marie José Kersten ^5^, Olalekan O. Oluwole ^6^, Armin Ghobad ^7^, Aaron P. Rapoport ^8^, Joseph P. McGuirk ^9^, John M. Pagel ^10^, Javier Muñoz ^11^, Umar Farooq ^12^, Tom Van Meerten ^13^, Patrick M. Reagan ^14^, Anna Sureda ^15^, Ian W. Flinn ^16^, Peter Vandenberghe ^17^, Kevin Song ^18^, Michael Dickinson ^19^, Monique C. Minnema ^20^, Peter A. Riedell ^21^, Lori A. Leslie ^22^, Sridhar Chaganti ^23^, Yin Yang ^24^, Simone Filosto ^24^, Marco Schupp ^24^, Christina To ^24^, Paul Cheng ^24^, Leo I. Gordon ^25^ and Jason R. Westin ^26^**

^1^ Moffitt Cancer Center, Tampa, FL, United States
^2^ Stanford University School of Medicine, Stanford University, Stanford, CA, United States
^3^ Dana-Farber Cancer Institute, Boston, MA, United States
^4^ Memorial Sloan-Kettering Cancer Center, New York, NY, United States
^5^ Amsterdam University Medical Centers, University of Amsterdam, Amsterdam, The Netherlands
^6^ Vanderbilt-Ingram Cancer Center, Nashville, TN, United States
^7^ Washington University School of Medicine, Washington University, Saint Louis, MO, United States
^8^ The Marlene and Stewart Greenebaum Cancer Center, University of Maryland School of Medicine, University of Maryland, Baltimore, MD, United States
^9^ University of Kansas Cancer Center, University of Kansas, Kansas City, KS, United States
^10^ Center for Blood Disorders and Stem Cell Transplantation, Swedish Cancer Institute, Seattle, WA, United States
^11^ Banner MD Anderson Cancer Center, Gilbert, AZ, United States
^12^ Department of Internal Medicine, University of Iowa, Iowa City, IA, United States
^13^ University Medical Center Groningen, University of Groningen, Groningen, The Netherlands
^14^ University of Rochester School of Medicine, University of Rochester, Rochester, NY, United States
^15^ Hematology Department, Institut Català d’Oncologia-Hospitalet, IDIBELL, Universitat de Barcelona, Barcelona, Spain
^16^ Sarah Cannon Research Institute and Tennessee Oncology, Nashville, TN, United States
^17^ University Hospitals Leuven, Leuven, Belgium
^18^ Division of Hematology, University of British Columbia and Leukemia/BMT Program of BC, Vancouver General Hospital, Vancouver, BC, Canada
^19^ Peter MacCallum Cancer Centre, Royal Melbourne Hospital, The University of Melbourne, Melbourne, Australia
^20^ University Medical Centers, University of Utrecht, Utrecht, The Netherlands
^21^ The University of Chicago Medical Center, University of Chicago, Chicago, IL, United States
^22^ John Theurer Cancer Center, Hackensack, NJ, United States
^23^ Centre for Clinical Haematology, University Hospitals Birmingham NHS Foundation Trust, Birmingham, United Kingdom
^24^ Kite, a Gilead Company, Santa Monica, CA, United States
^25^ Feinberg School of Medicine, Robert H. Lurie Comprehensive Cancer Center of Northwestern University, Northwestern University, Chicago, IL, United States
^26^ Department of Lymphoma and Myeloma, The University of Texas MD Anderson Cancer Center, Houston, TX, United States

**Background:** The standard of care (SOC) treatment (Tx) in the curative setting for patients (pts) with relapsed/refractory (R/R) large B-cell lymphoma (LBCL) after 1st-line (1L) chemoimmunotherapy (CIT) is high-dose therapy with autologous stem cell rescue (HDT-ASCT) if responsive to 2nd-line (2L) CIT; however, as many pts do not respond to or cannot tolerate 2L CIT, outcomes remain poor. Axi-cel is an autologous anti-CD19 chimeric antigen receptor (CAR) T-cell therapy approved for R/R LBCL after ≥2 prior systemic therapies.
**Purpose:** To report the results of the primary analysis of ZUMA-7, a global, randomized, Phase 3 trial of axi-cel vs. SOC in patients with 2L R/R LBCL.
**Methods:** Eligible pts ≥ 18 y with LBCL, refractory to or relapsed ≤12 mo of 1L CIT were randomized 1:1 to axi-cel or SOC (2–3 cycles of an investigator-selected, protocol defined, platinum-based CIT regimen followed by HDC-ASCT if CIT-responsive). Although there was no planned trial crossover between arms, pts not responding to SOC could receive CAR T-cell therapy off protocol. The primary endpoint was event-free survival (EFS: time to earliest date of disease progression, death from any cause, or new lymphoma Tx) by blinded central review. Key secondary endpoints, tested hierarchically, were objective response rate (ORR) and overall survival (OS; interim analysis); safety was also a secondary endpoint.
**Results:** As of 18 March 2021, 359 pts were randomized to axi-cel (*N* = 180) or SOC (*N* = 179). Overall, the median age was 59 y (range, 21–81; 30% ≥ 65 y), 74% of pts had primary refractory disease and 46% had high 2L age-adjusted IPI (2–3). While 170 (94%) axi-cel pts were infused, only 64 (36%) of SOC pts reached HDT-ASCT after 2L CIT. The primary endpoint of EFS was met (HR: 0.398; *p* < 0.0001). At 24.9 mo median follow-up, median EFS was significantly longer with axi-cel vs. SOC (8.3 mo vs. 2 mo, respectively), and Kaplan–Meier estimates of the 24-mo EFS rates were 41% vs. 16%. ORR and CR rates were higher with axi-cel vs. SOC (ORR: 83% vs. 50%; *p* < 0.0001; CR: 65% vs. 32%). Median OS, evaluated as a preplanned interim analysis, was not reached for axi-cel vs. 25.7 mo for SOC (HR: 0.708; *p* = 0.0159). In the SOC arm, 100 pts (56%) received commercially available or investigational CAR T-cell therapy off protocol as subsequent Tx. Grade ≥ 3 treatment-emergent adverse events occurred in 155 (91%) and 140 (83%) pts, and Tx-related deaths occurred in 1 and 2 pts in the axi-cel and SOC arms, respectively. In pts treated with axi-cel, grade ≥ 3 cytokine release syndrome (CRS) occurred in 11 (6%) and grade ≥ 3 neurologic events (NEs) occurred in 36 (21%). No grade 5 CRS or NEs occurred.
**Conclusions:** Axi-cel showed statistically significant improvement over SOC, with >4-fold greater median EFS, 2.5-fold greater EFS at 2 y, double the CR rate, and more than double the percentage of pts receiving definitive Tx. Safety of axi-cel was manageable and consistent with 3rd-line (3L) axi-cel therapy.

## Abstract 11 (Poster): Patient-Reported Outcomes in a Phase 3, Randomized, Open-Label Study Evaluating the Efficacy of Axicabtagene Ciloleucel (Axi-Cel) versus Standard of Care Therapy in Patients with Relapsed/Refractory Large B-Cell Lymphoma (ZUMA-7)

**Mahmoud Elsawy ^1^, Julio C. Chavez ^2^, Irit Avivi ^3^, Jean-François Larouche ^4^, Luciano Wannesson ^5^, Kate Cwynarski ^6^, Keren Osman ^7^, Kelly Davison ^8^, Jakob D. Rudzki ^9^, Saurabh Dahiya ^10^, Kathleen Dorritie ^11^, Samantha Jaglowski ^12^, John Radford ^13^, Franck Morschhauser ^14^, David Cunninghami ^15^, Alejandro Martin Garcia-Sancho ^16^, Dimitrios Tzachanis ^17^, Matthew L. Ulrickson ^18^, Reem Karmali ^19^, Natasha Kekre ^20^, Catherine Thieblemont ^21^, Gunilla Enblad ^22^, Peter Dreger ^23^, Ram Malladi ^24^, Namita Joshi ^25^, Wei-Jhih Wang ^25^, Caitlyn T. Solem ^25^, Julia Thornton Snider ^26^, Christina To ^26^ and Marie José Kersten ^27^**

^1^ Queen Elizabeth II Health Sciences Centre, Dalhousie University, Halifax, NS, Canada
^2^ Moffitt Cancer Center, Tampa, FL, United States
^3^ Tel Aviv Sourasky Medical Center, Sackler Faculty of Medicine, Tel Aviv University, Tel Aviv, Israel
^4^ Centre hospitalier universitaire de Québec, Hôpital de l’Enfant-Jésus, Québec City, QC, Canada
^5^ Istituto Oncologico della Svizzera Italiana, Bellinzona, Switzerland
^6^ Department of Haematology, University College London Hospital NHS Foundation Trust, London, United Kingdom
^7^ Icahn School of Medicine at Mount Sinai, New York, NY, United States
^8^ McGill University Health Centre, McGill University, Montreal, QC, Canada
^9^ University Clinic for Internal Medicine, The Medical University of Innsbruck, Innsbruck, Austria
^10^ University of Maryland Medical Center, University of Maryland, Baltimore, MD, United States
^11^ University of Pittsburgh Medical Center Hillman Cancer Center, University of Pittsburgh, Pittsburgh, PA, United States
^12^ The Ohio State University Comprehensive Cancer Center, Ohio State University, Columbus, OH, United States
^13^ The Christie NHS Foundation Trust, University of Manchester, Manchester, United Kingdom
^14^ Hematology Department, Lille University Hospital, Lille, France
^15^ The Royal Marsden NHS Foundation Trust, London, United Kingdom
^16^ Institute of Biomedical Research of Salamanca, Centro de Investigación Biomédica en Red de Cáncer, Salamanca University Hospital, Salamanaca, Spain
^17^ Department of Medicine, University of California, San Diego, La Jolla, CA, United States
^18^ Banner MD Anderson Cancer Center, Gilbert, AZ, United States
^19^ Division of Hematology and Oncology, Northwestern University, Chicago, IL, United States
^20^ The Ottawa Hospital, Ottawa, ON, Canada
^21^ Hemato-Oncology, Hôpital Saint-Louis, University of Paris, Paris, France
^22^ Uppsala University Hospital, Uppsala, Sweden
^23^ Department of Internal Medicine, University of Heidelberg, Heidelberg, Germany
^24^ Cambridge University Hospitals NHS Foundation Trust, Cambridge, CA, United Kingdom
^25^ OPEN Health, Bethesda, MD, United States
^26^ Kite, a Gilead Company, Santa Monica, CA, United States
^27^ AmsterdamUniversity Medical Center, University of Amsterdam, Cancer Center Amsterdam, Amsterdam, The Netherlands

**Background:** Outcomes are poor for patients with large B-cell lymphoma (LBCL) who relapse early or are refractory to first-line therapy. Furthermore, patients receiving second-line (2L) standard-of-care (SOC) therapy often report poor health-related quality of life (QoL; Lin V et al. J Clin Oncol. 2020; 38: e20070).
**Purpose:** To present the patient-reported outcomes (PROs) from ZUMA-7, a Phase 3, randomized, multicenter study comparing axi-cel (an autologous anti-CD19 chimeric antigen receptor [CAR] T-cell therapy) versus SOC as second-line treatment in relapsed/refractory LBCL.
**Methods:** PRO instruments, including the EORTC QLQ-C30 (cancer-specific 30-item questionnaire including global health status, functional, and symptom scales) and the EQ-5D-5L (a general questionnaire with 5 QoL domains plus a global assessment), were administered at baseline (prior to treatment), Day 50, Day 100, Day 150, Month 9, and every 3 months from randomization up to 24 months or time of event-free survival event (disease progression, death from any cause, or new lymphoma therapy), whichever occurred first. The QoL analysis set was defined as all patients who had a baseline PRO and ≥1 measure completed at Day 50, Day 100, or Day 150. Prespecified hypotheses for 3 PRO domains (EORTC QLQ-C30 Physical Functioning, EORTC QLQ-C30 Global Health Status/QoL, and EQ-5D-5L visual analog scale [VAS]) were tested. False discovery rate adjusted the *P* values across key endpoints; sensitivity analyses controlled for covariates and patterns of missingness. A clinically meaningful change was defined as 10 points for each EORTC QLQ-C30 score and 7 points for EQ-5D-5L VAS score. Exploratory analyses on other domains of EORTC QLQ-C30 and EQ-5D-5L were also performed.
**Results:** Of 359 patients enrolled in the ZUMA-7 study, 296 patients (165 axi-cel, 131 SOC) were included for analysis. There was a statistically significant (*p* < 0.0001) and clinically meaningful difference in mean change of scores from baseline at Day 100 in favor of axi-cel on all prespecified PRO domains (Figure 1). Furthermore, scores also significantly favored axi-cel over SOC for EORTC QLQ-C30 Global Health Status/QoL (*p* = 0.0124) and EQ-5D-5L VAS (*p* = 0.0004) at Day 150. For the pre-specified endpoints, the mean estimated scores for the axi-cel arm had numerically returned to or exceeded scores at baseline by Day 150 versus on or after Month 9 for the SOC arm. After Month 9, attrition (e.g., an EFS event) in the QoL analysis set was substantial, particularly in the SOC arm. Additional exploratory analyses of PRO endpoints on other domains of EORTC QLQ-C30 and EQ-5D-5L also showed improvements with axi-cel over SOC.
**Conclusions:** PROs from ZUMA-7 showed that treatment with axi-cel results in clinically meaningful improvement in QoL over SOC at Day 100 as measured by multiple validated PRO instruments. The data also suggest faster recovery to pretreatment QoL with axi-cel compared with SOC.

**Figure 1:**
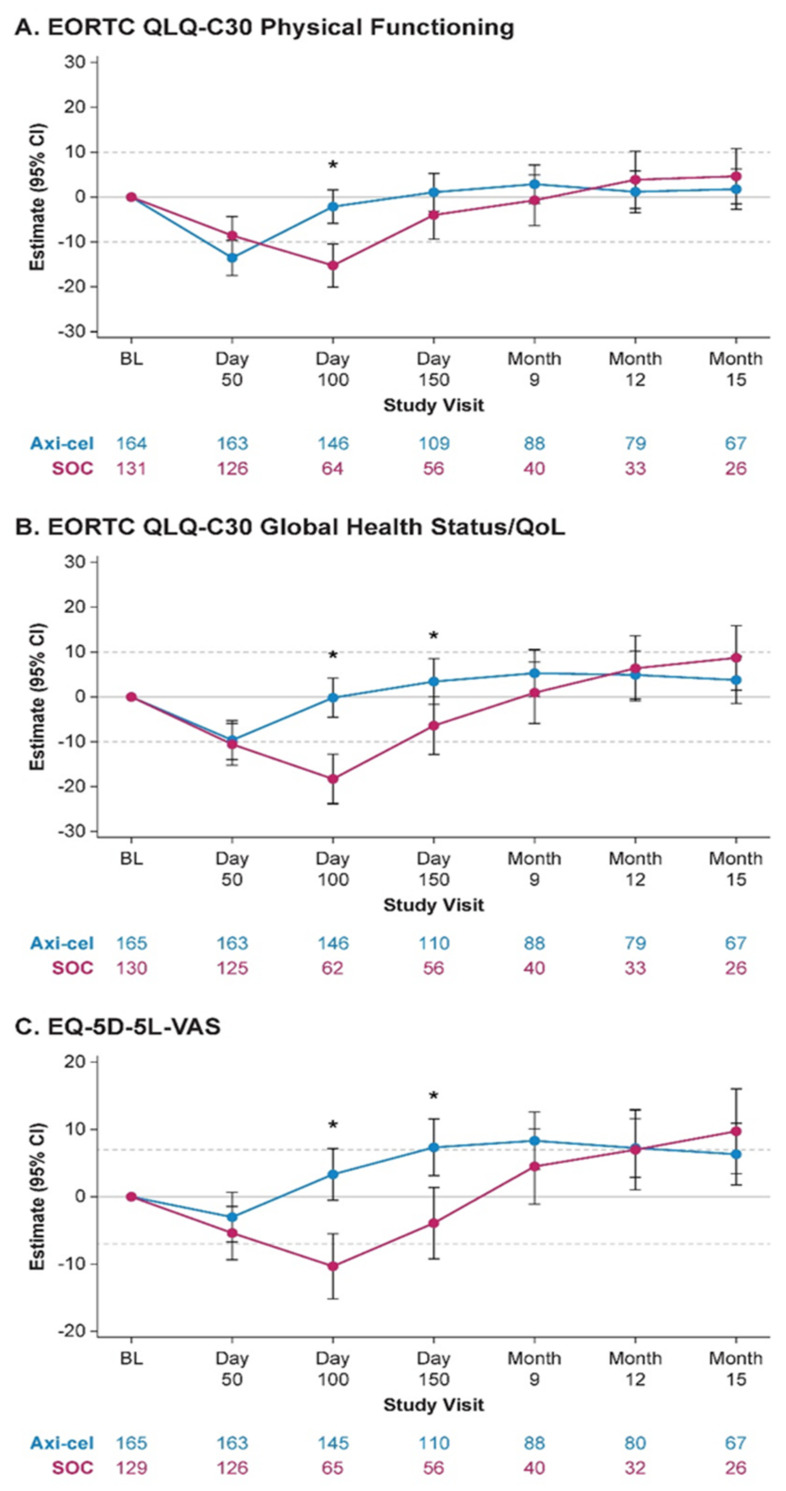
MMRM-based estimated changes from baseline by treatment arm. * *p* = 0.05; Axi-cel, axicabtagene ciloleucel; BL, baseline; CI, confidence interval; EORTC, European Organisation for Research and Treatment of Cancer; MMRM, mixed-effect model with repeated measures; SOC, standard of care.

## Abstract 12 (Poster): Efficacy Comparison of Tisagenlecleucel versus Standard of Care in Patients with Relapsed or Refractory Follicular Lymphoma

**Gilles Salles ^1^, Stephen J. Schuster ^2^, Martin Dreyling ^3^, Luca Fischer ^3^, John Kuruvilla ^4^, Piers E. M. Patten ^5,6^, Bastian von Tresckow ^7,8^, Sonali Smith ^9^, Ana Jiménez-Ubieto ^10^, Keith L. Davis ^11^, Carla Anjos ^12^, Jufen Chu ^12^, Jie Zhang ^12^, Chiara Lobetti Bodoni ^12^, Catherine Thieblemont ^13^, Nathan H. Fowler ^14^, Michael Dickinson ^15^, Joaquin Martínez-López ^10^, Yucai Wang ^16^, Brian K. Link ^17^**

^1^ Department of Medicine, Memorial Sloan Kettering Cancer Center, New York, NY, United States
^2^ Lymphoma Program, Abramson Cancer Center, University of Pennsylvania, Philadelphia, PA, United States
^3^ Department of Internal Medicine III, LMU Hospital, Munich, Germany
^4^ Division of Medical Oncology and Hematology, Princess Margaret Cancer Centre, Toronto, Canada
^5^ Comprehensive Cancer Centre, King’s College London, London, United Kingdom
^6^ Haematology, King’s College Hospital, London, United Kingdom
^7^ Department I of Internal Medicine, Medical Faculty, University Hospital Cologne, University of Cologne, Cologne, Germany
^8^ Department of Hematology and Stem Cell Transplantation, West German Cancer Center, University Hospital Essen, University of Duisburg-Essen, Essen, Germany
^9^ Section of Hematology/Oncology, The University of Chicago, Chicago, IL, United States
^10^ Hospital Universitario 12 de Octubre, Spanish National Cancer Research Center, Centro de Investigación Biomédica en Red Cáncer, Complutense University, Madrid, Spain
^11^ Health Economics, Research Triangle Institute Health Solutions, Research Triangle Park, Durham, NC, United States
^12^ Novartis Pharmaceuticals Corporation, East Hanover, NJ, United States
^13^ Hemato-Oncology Department, Saint Louis Hospital, Paris, France
^14^ University of Texas MD Anderson Cancer Center, University of Texas, Houston, TX, United States
^15^ Peter MacCallum Cancer Centre, Royal Melbourne Hospital, University of Melbourne, Melbourne, Australia
^16^ Division of Hematology, Mayo Clinic, Rochester, MN, United States
^17^ Department of Medicine, University of Iowa, Iowa City, IA, United States

**Background:** ELARA is an ongoing, single-arm, global, multicenter, phase II trial evaluating efficacy and safety of tisagenlecleucel (tisacel) in adult patients (pts) with relapsed/refractory follicular lymphoma (r/r FL). Tisacel demonstrated high response rates in pts with relapsed/refractory follicular lymphoma (r/r FL), with overall response rate (ORR) of 86% and complete response rate (CRR) of 66%. As ELARA did not include a comparator, an adjusted indirect treatment comparison (ITC) using patient-level data from a global retrospective cohort study was conducted.
**Purpose:** This study aimed to compare efficacy outcomes of tisacel from ELARA relative to standard of care (SOC).
**Methods:** As of 29 March 2021, 98 pts were enrolled in ELARA with a median follow-up of 15 months (M). SOC data were obtained from ReCORD-FL, a global retrospective cohort study of clinical outcomes in pts with r/r FL meeting the ELARA eligibility criteria who were treated per SOC at 10 academic centers in North America and Europe. In ReCORD-FL, 187 pts with ≥2 prior lines of treatment were included with a median follow-up from third line of 57 M. A case comparison analysis was performed for 97 ELARA apheresed pts and 143 ReCORD-FL pts using propensity score modelling and an adjusted ITC was performed to assess the effect of tisacel versus SOC by measuring CRR, ORR, progression-free survival (PFS), overall survival (OS), and time to next treatment (TTNT). A subgroup analysis of SOC pts with ≥1 eligible LoT initiated from 2014 (coinciding with the introduction of the Lugano response criteria and regulatory approval of idelalisib) was performed for all endpoints.
**Results:** Baseline characteristics (Figure 1) were well balanced after weighting. Treatment regimens observed for ReCORD-FL pts were: anti-CD20 antibody (Ab) plus alkylator (31.5% of pts), anti-CD20 Ab without alkylator (25.9%), alkylator without anti-CD20 Ab (17.5%), and regimens other than anti-CD20 Ab and alkylator (25.2%). After adjusting for differences in baseline variables, tisacel was associated with improvement over SOC in CRR (69.1% vs. 37.3%), ORR (85.6% vs. 63.6%), PFS, TTNT and OS (Figure 2), with a numerically higher 6 M PFS rate vs. SOC (85.3% vs. 66.5%), and higher 24 M OS rate (87.8% vs. 64.8%). There was an estimated 80% reduction in risk of death, 40% reduction in risk of progression for tisacel over SOC, and a 69% reduction in risk of death or requiring a new anticancer therapy (Figure 1). In the sub-analysis of SOC pts with lines of therapy initiated in or after 2014, the superiority of tisacel over SOC was confirmed in all efficacy outcomes (CRR: 69.1% vs. 30.5%; ORR: 85.6% vs. 58.8%; hazard ratios substantially < 1 for OS, PFS, TTNT).
**Conclusions:** These results suggest that tisacel has superior efficacy over SOC in r/r FL for all evaluated endpoints. Moreover, outcome parameters independent of response criteria (OS, TTNT) were also significantly better for tisacel vs. SOC.

**Figure 1:**
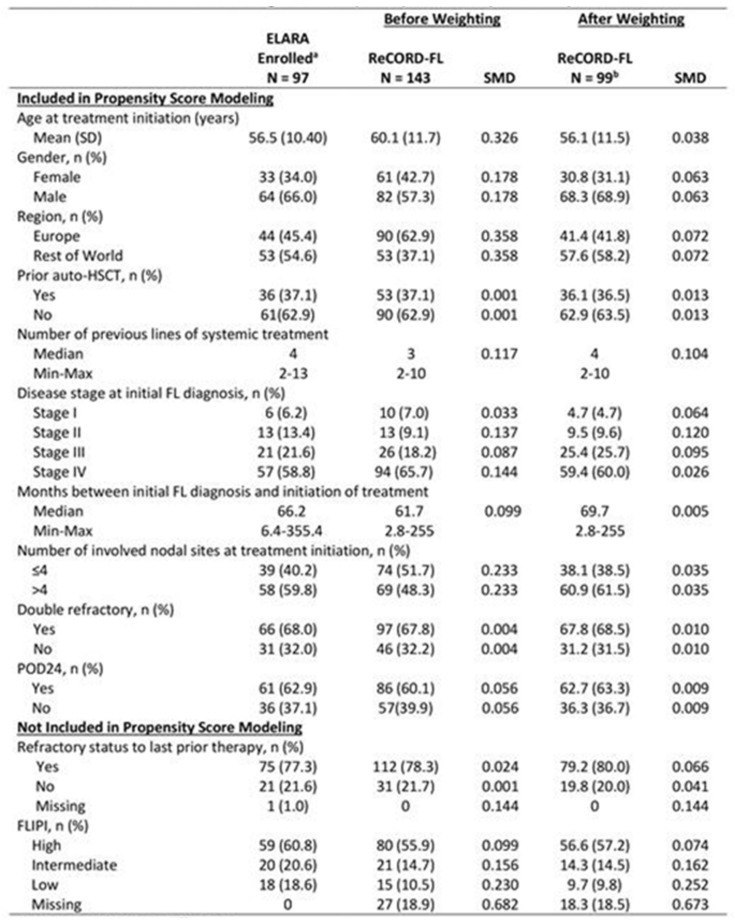
Baseline characteristics of tisagenlecleucel (ELARA) versus SOC (ReCORD-FL) Cohorts. SMD = standard mean difference. ^a^ Enrolled patients are those who met inclusion/exclusion criteria and had a leukapheresis product accepted for manufacturing, regardless of infusion status (only 1 enrolled patient was not infused). ^b^ Sample size after weighting (i.e., sum of weights) was 99 for the ReCORD study and effective sample size was 95.

**Figure 2:**
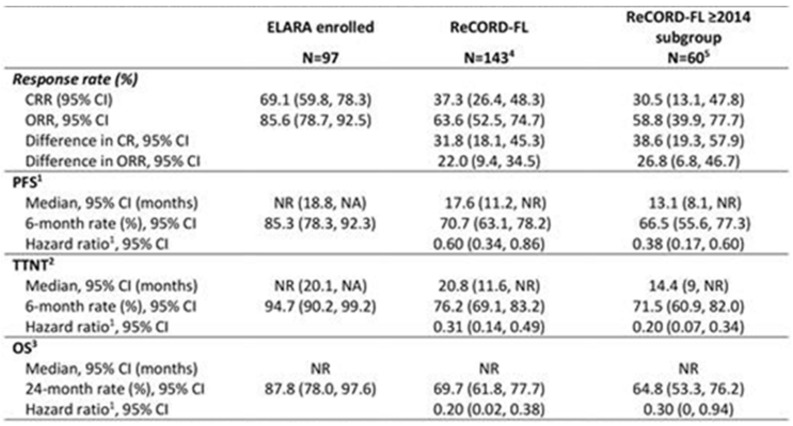
Indirect comparison of tisagenlecleucel (ELARA) versus SOC (ReCORD-FL) clinical outcomes. CI = confidence interval; NR = not reached; OS = overall survival; PFS = progression-free survival; TTNT = time to next treatment. Note: Outcomes across groups were compared using the weighting by odds method to adjust for differences in the following pre-specified baseline characteristics and prognostic factors: age, gender, geographic region, prior autologous hematopoietic stem cell transplant, number of previous LoTs, disease stage at initial FL diagnosis, time between diagnosis and treatment, extent of nodal involvement, double refractoriness at treatment start, and POD24 status. ^1^ PFS estimation considers new anti-cancer therapy as a progression event (in the absence of clinician-assessed progression or death before start of a new therapy). ^2^ TTNT estimation considers death as an event. ^3^ Hazard ratio calculated by weighted Cox proportional hazard model for indirect comparison between the ELARA and ReCORD-FL. ^4^ Sample size after weighting (i.e., sum of weights) was 99 and effective sample size was 95 for the main analysis. ^5^ Sample size after weighting (i.e., sum of weights) was 95 and effective sample size was 37 for the subgroup analysis.

## Abstract 13 (Poster): Canadian Practice Pattern on Management of Invasive Fungal Infections in Hematological Centres

**Jodi Faulkner ^1^, Shariq Haider ^2^, Gizelle Popradi ^3^, Jill Lacey ^4^, Marcela Roquim ^1^ and Emran Bashar ^1^**

^1^ AVIR Pharma, Blainville, QC, Canada
^2^ Juravinski Cancer Centre, McMaster University, Hamilton, ON, Canada
^3^ McGill University Health Center, McGill University, Montreal, QC, Canada
^4^ Saskatoon Cancer Centre, Saskatoon, SK, Canada

**Background:** Over the past decade, a substantial number of targeted therapies for hematological malignancies (HMs) have become available in Canada with improved disease-free survival and reduced toxicity. However, many of these new drugs exhibit moderate to severe drug–drug interactions with antifungal agents due to their extensive hepatic metabolism.
**Purpose:** This study aimed to (1) identify hematological anti-cancer agents and antifungal agents used in major Canadian centres, (2) describe the perceived association between these anti-cancer agents and the risk of invasive fungal infections (IFIs), and (3) describe the management of potential drug–drug interactions (DDIs).
**Methods:** A cross-sectional online survey containing 25 questions was conducted among 18 Infectious disease (ID), medical microbiology, hematology/oncology, and pharmacy specialists from 10 tertiary hospitals who manage patients with HMs that are at risk of developing IFIs. The results were analyzed, focusing on the HM agents that have been approved in the last ten years.
**Results:** Of the participants, the majority (67%) practice in hematology (physicians and pharmacists), but all respondents’ specialties are involved in IFI management. Across Canada, the perception is that candidiasis is the most commonly seen IFI, followed by aspergillosis and mucormycosis. Fluconazole, voriconazole, and posaconazole are among the most commonly used antifungals in these centres, with voriconazole identified by most (83%) as a drug requiring therapeutic drug monitoring. Posaconazole is the most frequently used prophylactic antifungal (61%) in acute myeloid leukemia (AML) patients, while fluconazole is the most frequently used agent in allogeneic or autologous stem cell transplant recipients and acute lymphoblastic leukemia (ALL) patients. In their respective centres, respondents can access hematological agents not included in protocols close to 90% of the time. Among the recently approved HM therapies, more than 50% of respondents reported “commonly” using gilteritinib, ibrutinib, midostaurin, ruxolitinib, blinatumomab, brentuximab, and venetoclax. Of these agents, venetoclax and ibrutinib were identified by at least 50% of the respondents as associated with an elevated incidence of IFI. Ninety-four percent of respondents consult their pharmacists to predict DDI, and the most common (94%) technique to manage DDI is to empirically adjust doses.
**Conclusions:** This initial data set shows substantial variability in the usage of hematological and antifungal agents, therapeutic monitoring of agents, and perceived infection risk of new HM agents among Canadian Centres. Expanding the scope of this survey to include more sites would ensure greater reliability and validity to the observations and support the development of consensus recommendations around managing specific DDI when treating fungal infections in high-risk hematological patients.

## Abstract 14 (Poster): Upfront Haploidentical Bone Marrow Transplant for Patients with Severe Aplastic Anemia—Single Centre Experience

**Vered Stavi, Wilson Lam, Arjun D. Law, Armin Gerbitz, Ivan Pasic, Auro Viswabandya, Fotios V. Michelis, Dennis Kim, Jonas Mattsson, Jeffrey H. Lipton and Rajat Kumar**

Hans Messner Allogeneic Blood and Marrow Transplantation Program, Princess Margaret Cancer Center, Toronto, ON, Canada

**Background**: In adult patients with severe aplastic anemia (SAA) first line treatment with bone marrow transplant (BMT) is recommended only if patients are young and have a matched sibling donor. For others, immunosuppressive therapy (IST) is considered first line, while matched unrelated donor (MUD) transplants are considered second line. Related haploidentical donor (HID) transplants for SAA are still considered experimental (Killick SB et al. BJ Haem 2016). Upfront HID transplants for SAA are rare. At Princess Margaret we have performed three upfront HID bone marrow transplants (BMT) for SAA in recent years with success.
**Purpose:** To share our experience of upfront HID BMT for SAA. This is especially relevant for ethnic minorities, who often lack a MUD donor.
**Methods:** Retrospective study. We collected data from our transplant database of all transplants for SAA. There were three patients who underwent upfront HID transplant. Their informed consent was obtained and details reviewed from electronic patient records.
**Results:** Number of patients: 3 (Figure 1)
Protocol used: Baltimore protocol [1]:
  (a) Conditioning: Anti-thymocyte globulin (ATG)—Fludarabine (FLU)—Cyclophosphamide (CY)—Total Body Irradiation (TBI) 400 cGy.
  (b) Graft-vs.-host disease (GVHD) prophylaxis with: PTCY-Cyclosporine and MMF.
Graft source: bone marrow. Median age at diagnosis of SAA: 24 y (range 20–29). Median time from diagnosis to transplant: 103 days (range 62–177). All patients engrafted. Median time to neutrophils engraftment was 21.3 days (range 20–22). No patient had graft failure. One patient had poor graft function that resolved after Eltrombopag treatment. Only one patient experienced aGVHD (grade 2 skin).
  **Conclusions:** Haploidentical SCT as upfront therapy was an effective and safe option for three SAA patients, with favorable outcomes. This type of transplant can be done in a very timely manner in young patients with SAA who lack a MSD, as first line therapy instead of IST.

**Figure 1:**
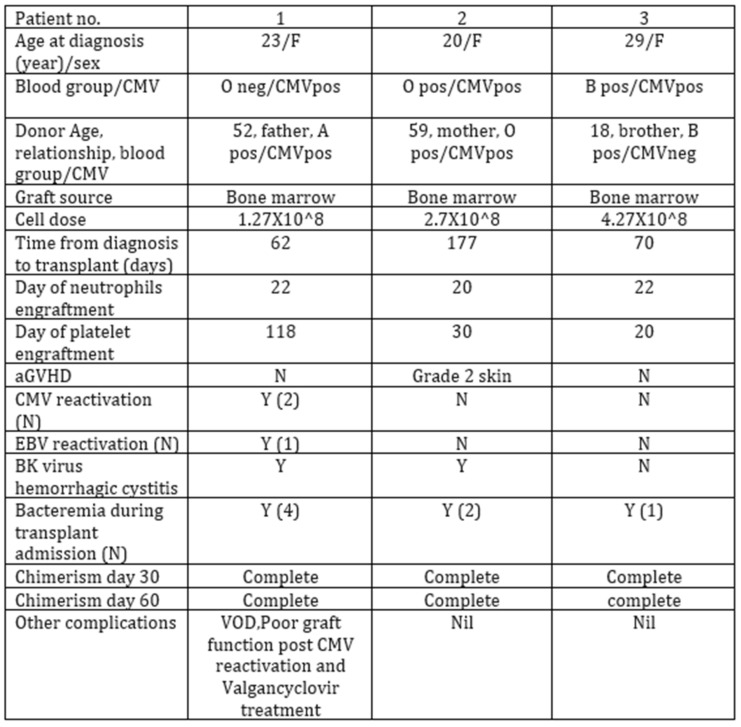
Transplant characteristics.

**References:**
  1. DeZern, A.E.; Zahurak, M.L.; Symons, H.J.; Cooke, K.R.; Rosner, G.L.; Gladstone, D.E.; Huff, C.A.; Swinnen, L.J.; Imus, P.; Borrello, I.; et al. Haploidentical BMT for severe aplastic anemia with intensive GVHD prophylaxis including posttransplant cyclophosphamide. *Blood Adv.* **2020**, *4*, 1770–1779. https://doi.org/10.1182/bloodadvances.2020001729.

## Abstract 15 (Oral): CLIC-1901 CAR-T Cells for the Treatment of Patients with Relapsed/Refractory CD19 Positive Hematologic Malignancies (CLIC-01 Study) (Award Recipient—Clinical Trials/Observations)

**Natasha Kekre ^1,2^, Kevin A. Hay ^3,4,5^, John R. Webb ^6^, Miruna Balasundaram ^7^, Mhairi Sigrist ^7^, Anne-Marie Clement ^1,2^, Julie S. Nielsen ^6^, Scott Brown ^7^, Manoj M. Lalu ^1^, Dean A. Fergusson ^1^, John C. Bell ^8,9^, Harold Atkins ^2,8,10^, Brad H. Nelson ^6,11,12^ and Robert A. Holt ^7,12,13^**

^1^ Clinical Epidemiology Program, Ottawa Hospital Research Institute, Ottawa, ON, Canada
^2^ Division of Hematology, Department of Medicine, The Ottawa Hospital, Ottawa, ON, Canada
^3^ Division of Hematology, Department of Medicine, University of British Columbia, Vancouver, BC, Canada
^4^ Terry Fox Laboratory, British Columbia Cancer Research Institute, Vancouver, BC, Canada
^5^ Leukemia and Bone Marrow Transplant Program of British Columbia, Vancouver, BC, Canada
^6^ Trev and Joyce Deeley Research Centre, British Columbia Cancer, Victoria, BC, Canada
^7^ Canada’s Michael Smith Genome Sciences Centre, British Columbia Cancer Research Institute, Vancouver, BC, Canada
^8^ Center for Innovative Cancer Therapeutics, Ottawa Hospital Research Institute, Ottawa, ON, Canada
^9^ Department of Biochemistry, Microbiology and Immunology, University of Ottawa, Ottawa, ON, Canada
^10^ Department of Cellular Molecular Medicine, University of Ottawa, Ottawa, ON, Canada
^11^ Department of Biochemistry and Microbiology, University of Victoria, Victoria, BC, Canada
^12^ Department of Medical Genetics, University of British Columbia, Vancouver, BC, Canada
^13^ Department of Molecular Biology & Biochemistry, Simon Fraser University, Burnaby, BC, Canada

**Background:** CAR-T cell therapy has proven effective in treating adult patients with relapsed/refractory CD19 positive B cell malignancies that have failed standard therapies, but access to commercial CD19 CAR-T remains limited in Canada.
**Purpose:** We designed a multicenter, two-stage, single-arm, open-label early phase study to determine the safety and efficacy of Canadian-made CD19 CAR-T cells (*CLIC-1901*) in participants with CD19^+^ ALL, CLL and NHL.
**Methods:** The anti-CD19 CAR transgene, developed in Vancouver (BC Cancer), contained a 4-1BB costimulatory domain. Lentivirus containing the transgene was produced at the Ottawa Biotherapeutics Manufacturing Center. Peripheral blood mononuclear cells were collected by leukapheresis in Ottawa and Vancouver and shipped to the CAR-T cell manufacturing facility in Victoria. T cells were selected, activated, transduced, and expanded using a GMP compliant semi-automated, closed process using the Miltenyi Prodigy. Final CLIC-1901 product was tested for identity, potency, purity, and sterility, and only infused if release criteria met. Participants underwent lymphodepletion with fludarabine (40 mg/m^2^/d × 3) and cyclophosphamide (500 mg/m^2^/d × 2), prior to infusion of >1 × 10^6^ CAR expressing cells per kilogram of body weight (maximum 2 × 10^8^ total CAR expressing cells) non-cryopreserved CLIC-1901.
**Results:** Of 48 patients screened for eligibility, 35 were enrolled. 5 enrolled participants did not receive CLIC-1901 due to manufacturing failures early after protocol launch (*n* = 2, resolved with first protocol amendment), severe myocarditis before lymphodepletion (*n* = 1), and death before infusion (*n* = 2). 30 participants received CLIC-1901 CAR-T therapy: 21 males (70%), median age 66 (range 18–75). The median number of prior therapies was 3 (range 2–6). 13 (43%) patients had failed a stem cell transplant (allogeneic (*n* = 5), autologous (*n* = 6), both (*n* = 2)). The disease indication was DLBCL (*n* = 10), MCL (*n* = 8), transformed DLBCL (*n* = 4), ALL (*n* = 5), follicular lymphoma (*n* = 1), Richter’s transformation (*n* = 1) and plasmablastic lymphoma (*n* = 1). The time from enrollment to CLIC-1901 infusion was a median of 20 days (range 15–48). The median CLIC-1901 dose infused was 2.3 × 10^6^ CAR-T cells/kg (range 1.3 × 10^5^–3.6 × 10^6^/kg). Toxicity included CRS (grade 1 *n* = 9, grade 2 *n* = 7, grade 3 *n* = 1, and grade 5 *n* = 1), at median onset of 1.5 days after CLIC-1901 infusion (range 0–9 days). ICANS occurred in 2 participants (grade 2 *n* = 1, and grade 4 *n* = 1). At a median follow-up of 4 months (IQR 4–7), the median progression-free survival was 5 months (95% CI 4-not estimable). Figure 1 shows patient level data.
**Conclusions:** This is the first trial of Canadian-made CAR-T cells and demonstrates that Canadian manufacturing of vector, virus, and T cells is feasible within the academic sphere in Canada. Our preliminary results indicate that CLIC-1901 is safe and tolerable. Longer follow-up and more patients are needed to determine overall efficacy.

**Figure 1:**
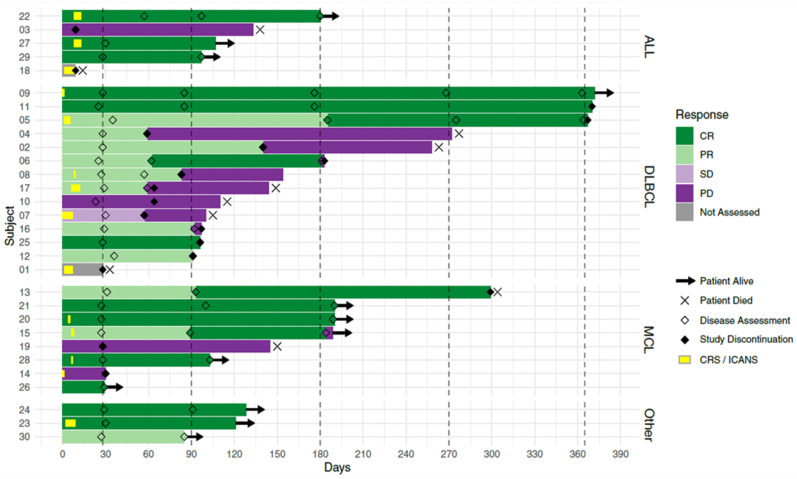
Swimmer’s plot of first 30 patients treated with CLIC-1901 from time of cell infusion.

## Abstract 16 (Poster): The Efficacy and Safety of Low-Dose Inotuzumab Ozogamicin in Patients with Relapsed or Refractory Acute Lymphoblastic Leukemia: Interim Results of a Phase 4 Study

**Muhit Özcan ^1^, Ryan D. Cassaday ^2^, Pawan Singh ^3^, Ewa Zarzycka ^4^, Xin Zhang ^5^, Eric Nègre ^6^, Erik Vandendries ^7^ and Fevzi Altuntas ^8^**

^1^ Ankara University Cebeci Hospital, Ankara, Turkey
^2^ Fred Hutchinson Cancer Research Center, Seattle Cancer Care Alliance, University of Washington, Seattle, WA, United States
^3^ Artemis Hospital, Gurugram, India
^4^ Klinika Hematologii i Transplantologii, Uniwersyteckie Centrum Kliniczne, Gdansk, Poland
^5^ Pfizer Inc., Shanghai, China
^6^ Pfizer Inc., New York, NY, United States
^7^ Pfizer Inc., Cambridge, MA, United States
^8^ Dr. Abdurrahman Yurtaslan Ankara Oncology Training and Research Hospital, Ankara, Turkey

**Background:** Inotuzumab ozogamicin (InO) is approved (US, EU, CA) for relapsed/refractory acute lymphoblastic leukemia (R/R ALL). Patients (pts) receiving the approved starting dose (1.8 mg/m^2^/cycle, 3 divided doses) in the phase 3 INO-VATE trial had a higher rate of remission and were more likely to proceed to hematopoietic stem cell transplantation (HCT) vs. pts in the chemotherapy arm but were more likely to experience post-HCT hepatic sinusoidal obstruction syndrome (SOS). It is not known whether a lower dose of InO would improve safety and reduce the likelihood of post-HCT SOS and whether this would impact efficacy.
**Purpose:** The phase 4 study (NCT03677596; FDA post-marketing requirement) aims to assess efficacy/safety of 2 dose levels of InO in adults with R/R ALL who are eligible for HCT and have a higher risk of post-HCT SOS.
**Methods:** The ongoing open-label study will involve ~102 pts. In an initial run-in phase (Simon 2-stage design), 22 pts (7 stage 1, 15 stage 2) received a starting dose of 1.2 mg/m^2^/cycle in 3 divided doses. To proceed to the randomized phase, this dose needs to show acceptable efficacy during run-in: rate of complete remission (CR)/CR with incomplete hematologic recovery (CRi) needs to be ≥3/7 in stage 1, ≥10/22 in stage 2, with minimal residual disease (MRD) negativity ≥ 7/22. In the randomized phase, up to 80 pts will receive InO at 1.2 or 8 mg/m^2^/cycle. Pts are followed for ≥2 years post-randomization.
**Results:** In the run-in phase, 22 pts received InO at 1.2 mg/m^2^/cycle: median duration, 6 (range 0.1–22.7) wks; median 2 (1–6) treatment cycles; median age, 46 (21–67) y; male, 54.5%. Risk factors at baseline for post-HCT SOS: previous HCT, 31.8%; salvage ≥ 2, 68.2%; age ≥ 55, 22.7%; prior/ongoing hepatic disease, 27.3%. Half of pts discontinued treatment (11/22; death, *n* = 3; progressive disease, *n* = 7; relapse, *n* = 1).
In stage 1, 3/7 pts achieved CR/CRi (2 CR, 1 CRi). In stage 2, half of pts achieved CR/CRi; >70% of these were MRD negative (Figure 1). Almost a third of pts proceeded to HCT (31.8%). Three pts died during, and 10 after, treatment (4 in disease follow-up, 6 in long-term follow-up; disease progression, *n* = 7; AEs not related to study treatment, *n* = 4; hepatic SOS, *n* = 1; sepsis, *n* = 1).
Treatment-emergent AEs (TEAEs, any grade) occurred in 90.9% (20/22) of pts. The most common grade ≥ 3 TEAEs included hematologic disorders and infections (Figure 2). Of pts who proceeded to HCT, 28.5% (2/7) had post-HCT SOS: 1 grade 5 (pt with ongoing or prior hepatic disease); 1 grade 2 (pt in salvage ≥ 2 with prior HCT). There were 5 additional grade 5 AEs (disease progression, *n* = 3; infections, *n* = 2). Two pts (9.1%) discontinued due to AEs (tumor lysis syndrome, fungal infection).
**Conclusions:** At a starting dose of 1.2 mg/m^2^/cycle, InO showed acceptable efficacy, with half of pts achieving remission and >70% of those being MRD negative. No new safety signals were identified. The study is proceeding to the randomized phase (*N* = 91 as of July 2021).

**Figure 1:**
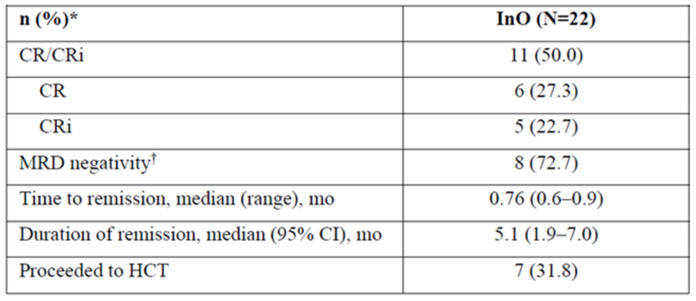
Efficacy of inotuzumab ozogamicin at a starting does of 1.2 mg/m^2^/cycle. * Unless otherwise noted; ^†^ Among patients achieving CR/CRi (N = 11). CR = complete remission; CRi = complete remission with incomplete hematologic recovery; HCT = hematopoietic cell transplantation; mo = months; InO = inotuzumab ozogamicin; MRD = minimal residual disease, assessed using flow cytometry at Navigate, Carlsbad, CA, United States.

**Figure 2:**
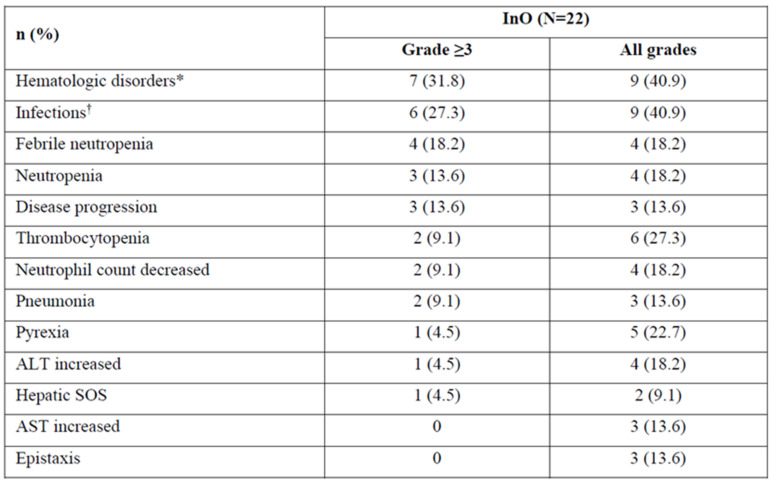
TEAEs occurring in ≥10% patients and AEs of special interest with inotuzumab ozogamicin at a starting dose of 1.2 mg/m^2^/cycle. * Includes the system organ class blood and lymphatic system disorders; ^†^ Includes the system organ class infections and infestations. AE = adverse event; ALT = alanine aminotransferase; AST = aspartate aminotransferase; SOS = sinusoidal obstruction syndrome; TEAE = treatment–emergent adverse event.

## Abstract 17 (Poster): Hemophagocytic Lymphohistiocytosis (HLH) after Allogeneic HSCT for Relapsed DLBCL in the Setting of STK4 Immunodeficiency

**Anath C. Lionel ^1,2^, Donna Wall ^3^, Ahmed Naqvi ^3^, Chaim Roifman ^3^ and Tobias Berg ^1,2^**

^1^ Department of Oncology, Juravinski Cancer Centre, McMaster University, Hamilton, ON, Canada
^2^ Centre for Discovery in Cancer Research, McMaster University, Hamilton, ON, Canada
^3^ Department of Pediatrics, Faculty of Medicine, University of Toronto, Toronto, ON, Canada

**Background**: HLH is a hyper-inflammatory condition, which if left untreated can result in multi-organ failure and high mortality. While HLH has been reported in patients who have received hematopoietic stem cell transplantation (HSCT), its diagnosis can be challenging in this setting because of clinical features overlapping with other common post-HSCT complications such as sepsis, recurrent malignancy, and graft versus host disease (GVHD).
**Purpose:**
  1. To illustrate the diagnosis and management of HLH in the post-HSCT setting.
  2. First report of HLH in the context of STK4 deficiency, a rare genetic, combined B and T cell immunodeficiency.
**Methods:** Case Report.
**Results:** The patient is a 22-year-old Syrian-Canadian male with homozygous frameshift *STK4* mutations (NM_006282:exon9:c.1103delT:p.M368fs); his parents are both heterozygous carriers. Complications related to his immunodeficiency include ITP, eczema, corneal erosions, recurrent mouth sores, and HPV warts. In 2018, he was diagnosed with diffuse large B-cell lymphoma (DLBCL) based on a biopsy of liver lesions. He was treated with 6 cycles of R-CHOP followed by a PET scan showing complete metabolic response. In 2020, he was diagnosed with relapsed DLBCL and was treated with salvage chemotherapy with evidence of remission. After a discussion involving the immunology, lymphoma, and transplant teams, he underwent an allogeneic HSCT in June 2020 as a potentially curative option for both his relapsed lymphoma and primary immunodeficiency. The donor was his sister, who was a carrier of the heterozygous STK4 mutation. Post-HSCT complications included biopsy-proven GVHD of the gut in June 2020 and of the skin in January 2021 as well as CMV and adenovirus infections.
He was re-admitted in September 2021 with fever and maculopapular rash involving bilateral hands and feet. Extensive workup did not reveal any signs of underlying infectious etiology, recurrence of lymphoma, or marrow graft failure. He met diagnostic criteria for HLH including fever, significantly elevated ferritin (peak value 243,000 ug/L), elevated triglycerides, presence of hemophagocytes in bone marrow aspirate, and reduced NK cell degranulation activity. Therapy for HLH was initiated with dexamethasone followed by the addition of Anakinra and Ruxolitinib. He experienced clinical remission and improvement in lab parameters including the normalization of CRP and down-trending of ferritin.
**Conclusions:** This case illustrates the need to consider HLH as a differential in allogeneic HSCT recipients presenting with febrile illness of unclear etiology. Screening for HLH using serum ferritin would be particularly important in those patients with underlying immunodeficiency and/or prior histories of GVHD, CMV or other viral reactivation. Timely recognition and therapy initiation for HLH would be important given its associated high mortality.

## Abstract 18 (Poster): Bortezomib Maintenance after Upfront Allogeneic Transplantation in Young or High-Risk Myeloma Patients Leads to Less Chronic GVHD and Immunosuppression

**Jean-Sébastien Claveau ^1,2^, Richard LeBlanc ^2^, Imran Ahmad ^2^, Jean-Sébastien Delisle ^2^, Nadia M. Bambace ^2^, Léa Bernard ^2^, Sandra Cohen ^2^, Thomas Kiss ^2^, Sylvie Lachance ^2^, Denis Claude Roy ^2^, Guy Sauvageau ^2^, Olivier Veilleux ^2^ and Jean Roy ^2^**

^1^ Division of Hematology, Mayo Clinic, Rochester, MN, United States
^2^ Division of Hematology, Oncology and Transplantation, Maisonneuve-Rosemont Hospital, Université de Montréal, Montreal, QC, Canada

**Background:** Allogeneic (allo) hematopoietic cell transplant (HCT) has curative potential in myeloma (MM) but remains hampered by high rates of relapse and chronic (c) GVHD. In a recent prospective phase II study in young or high-risk newly diagnosed MM using bortezomib (BTZ) maintenance after tandem auto/allo HCT targeted at decreasing relapse (LeBlanc R, BMT 2021), we observed a 20% lower incidence of cGVHD compared to our historical cohort (Ahmad I, BMT 2016).
**Purpose:** We sought to further explore the impact of BTZ maintenance on the incidence and severity of cGVHD in patients who received maintenance or not.
**Methods:** Using 2015 NIH criteria, we retrospectively reviewed the incidence and organ distribution of cGVHD and duration of systemic immunosuppression in patients receiving BTZ 1.3 mg/m^2^ once every 2 weeks for one year after allo HCT or not. After auto HCT, MM patients from both cohorts received an outpatient nonmyeloablative conditioning followed by G-CSF mobilized donor stem cells. GVHD prophylaxis consisted of mycophenolate mofetil and tacrolimus weaned by D + 100 (sibling donors) or D + 180 (unrelated donors). Cumulative incidences of cGVHD were estimated using competing-risk methods.
**Results:** Between 2014 and 2018, 41 consecutive patients receiving BTZ maintenance were compared to 57 patients who did not. Baseline characteristics showed no difference except that patients in the BTZ group had younger (40 years vs. 52 years) and more unrelated donors (61% vs. 12%). Incidence of grade II-IV acute GVHD at D + 120 was similar in BTZ and controls (12.2% vs. 12.3%, *p* = 0.518). At 2 years, incidences of overall (61.0% vs. 84.2%, *p* = 0.001) and moderate/severe cGVHD (44.7% vs. 66.7%, *p* = 0.001, Figure 1) were significantly lower in BTZ recipients. In univariate analysis, overall mouth (56% vs. 79%, *p* = 0.026), skin (34% vs. 56%, *p* = 0.041) and liver (32% vs. 54%, *p* = 0.039) involvement were less frequent in BTZ patients. The cumulative incidence of prednisone initiation at 5 years was 42.2% in BTZ and 78.3% in no BTZ recipients (*p* < 0.001). The cumulative incidence of tacrolimus resumption at 5 years was also lower in BTZ than in controls (30.1% vs. 73.6%, *p* < 0.001). Probability of being alive and off systemic immunosuppressants at 3 years were 73% for BTZ patients vs. 42% for controls (*p* < 0.001, Figure 2). NRM at 2 and 5 years were 4.9% and 8.8% in BTZ recipients vs. 1.8% and 5.4% in no BTZ recipients (*p* = 0.567). We observed no impact of BTZ on 5-year OS (82.9% vs. 83.4%, *p* = 0.938), PFS (43.1 vs. 54.2%, *p* = 0.208) or relapse (48.1% vs. 40.5%, *p* = 0.321) in patients receiving or not BTZ.
**Conclusions:** BTZ maintenance led to a significant reduction in incidence and severity of cGVHD with shorter duration of systemic immunosuppressants but without impacting survival or relapse. BTZ maintenance should be considered as a valid option in MM receiving allo HCT.

**Figure 1:**
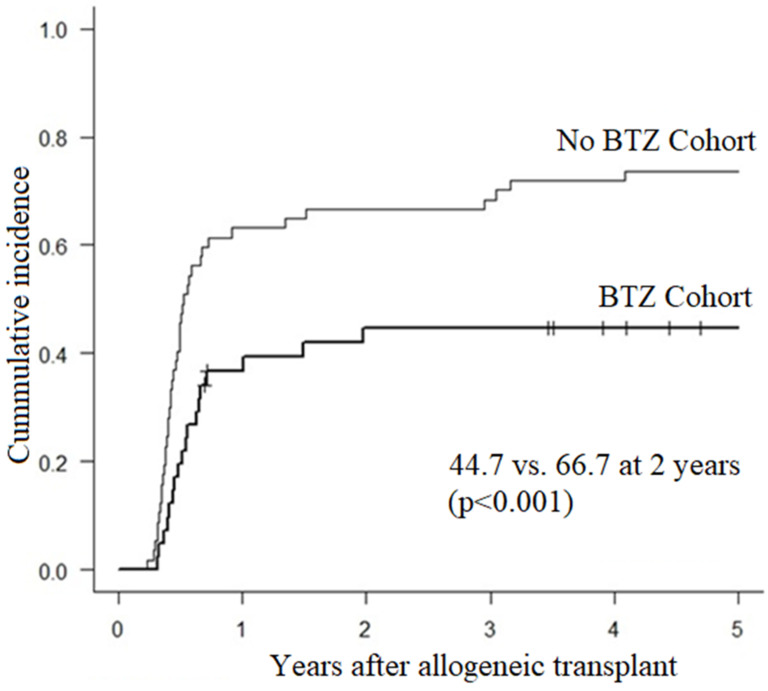
Incidence of moderate to severe cGVHD after tandem auto/allo HCT in recipients of BTZ or not.

**Figure 2:**
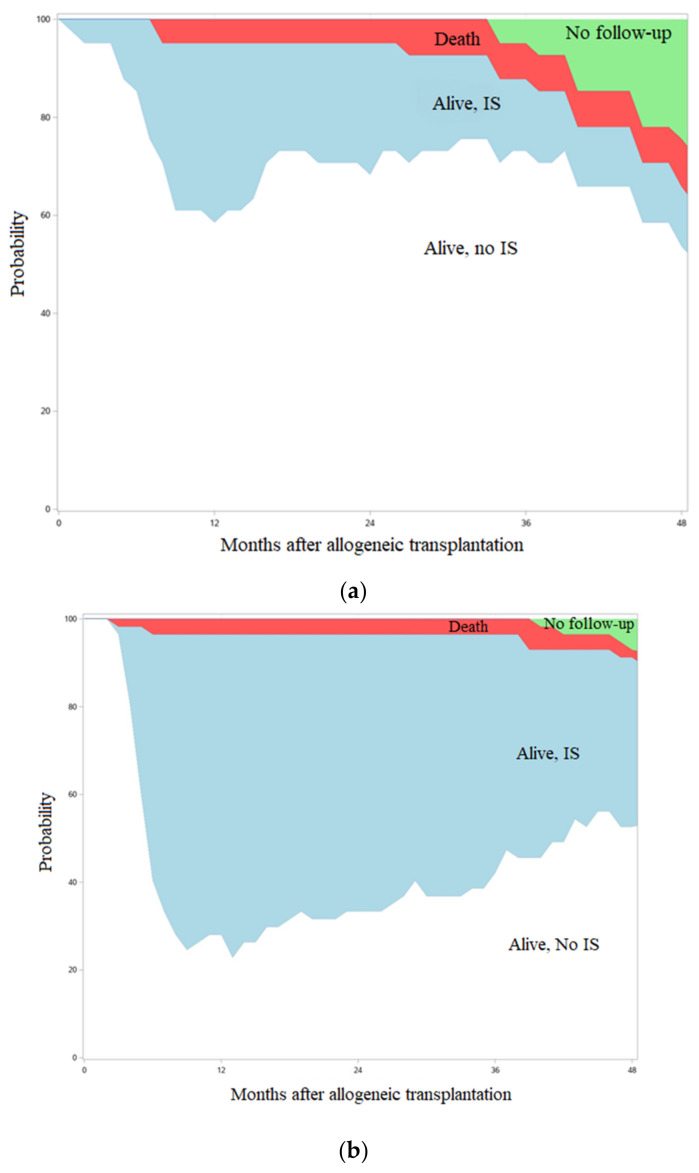
Systemic immunosuppression burden after transplant in patients with (**a**) or without (**b**) BTZ maintenance.

## Abstract 19 (Poster): Myeloablative Conditioning and Peripheral Blood Stem Cells from Haploidentical Donors Offers Comparable Outcomes to Matched Donors in Allogeneic Transplantation for Haematological Malignancies

**Kristjan Paulson ^1,2^, Gizelle Popradi ^3^, Donna Wall ^4^, Oliver Bucher ^2^, Geoffrey D. E. Cuvelier ^1^, Pauline Lambert ^2^, Grace Musto ^2^, Erin Richardson ^2^, Matthew D. Seftel ^5^, David Szwajcer ^1^ and Andrew Daly ^6^**

^1^ Department of Internal Medicine, University of Manitoba, Winnipeg, MB, Canada
^2^ CancerCare Manitoba, Winnipeg, MB, Canada
^3^ Stem Cell Transplant Program, McGill University Health Center, Montreal, QC, Canada
^4^ Department of Immunology, University of Toronto, Toronto, ON, Canada
^5^ Canadian Blood Services, Ottawa, ON, Canada
^6^ Department of Medicine, University of Calgary, Calgary, AB, Canada

**Background:** For patients without matched donors, the use of haploidentical donors with post-transplant cyclophosphamide (PTCy) is often considered. Most studies evaluating this combination have used bone marrow (BM) grafts with reduced intensity conditioning (RIC). The safety and relative efficacy of haploidentical donor transplants performed using peripheral blood stem cells (PBSC) and myeloablative conditioning (MAC) regimens remains less clear.
**Methods**: We conducted a prospective cohort study at three transplant centres in Canada using the Cell Therapy Transplant Canada registry as a data collection tool. Patients without a matched donor undergoing transplant for a hematologic malignancy were eligible. A CD34 dose of 3–8 × 10^6^/kg was infused following a MAC regimen of fludarabine (200 mg/m^2^) and busulfan (12.8 mg/kg) (Flu/Bu). At the discretion of the investigator, low dose total body irradiation could be added (200 or 400 cGy). GVHD prophylaxis consisted of PTCy (50 mg/kg on days +3 and +4) in combination with MMF (days 5–35) and tacrolimus (days 5–100). To determine how outcomes compared to matched sibling donors (MSD) and matched unrelated donors (MUD), a comparison was done with controls from the CTTC registry. To provide a more homogenous cohort, this analysis was restricted to patients undergoing transplant for acute myeloid leukemia (AML).
**Results:** Thirty-four patients were accrued in the prospective cohort, with diagnoses of acute myeloid leukemia (*n* = 24), acute lymphoblastic leukemia (*n* = 7), or myelodysplastic syndrome (*n* = 3). The median age was 55 years (range 10–70). Of these, 52% were men, with a median KPS of 90 (range 80–100). The median HCT-CI was 0, with 4 patients having a HCT-CI of greater than 3. The protocol was well tolerated, with a cumulative incidence of non-relapse mortality of 19% at 2 years and 1 case of veno-occlusive disease (which resolved with therapy). The cumulative incidence of relapse at 2 years was 22%. The incidence of grade III/IV acute GVHD was 6%, and chronic extensive GVHD was 29%. Overall survival at 2 years was 63%. Based on the success of this protocol, it was adapted as the standard for patients undergoing haploidentical transplant at these centres, and an additional 20 patients with AML were transplanted. These 44 patients with AML (24 on the original study, 20 in the expansion cohort) were compared to 128 MSD transplants and 267 MUD transplants done with similar transplant characteristics (AML, MAC Flu/Bu, and PBSC), with data obtained from the CTTC registry. In multivariate analysis, adjusting for age, HCT-CI, KPS, and disease risk (by ELN), no difference in overall survival (OS), disease free survival (DFS), relapse rate, or non-relapse mortality was found between donor sources (Figure 1). Older age was a predictor of inferior DFS and NRM, HCT-CI was associated with NRM, and high-risk disease was associated with a higher relapse rate.
**Conclusions:** The use of PBSCs from haploidentical donors with MAC appears to be safe and effective, with outcomes comparable to MSD and MUDs, and low rates of acute and chronic GVHD. The feasibility of this combination provides further evidence supporting the use of haploidentical donors outside the previously studied RIC/BM setting.

**Figure 1:**
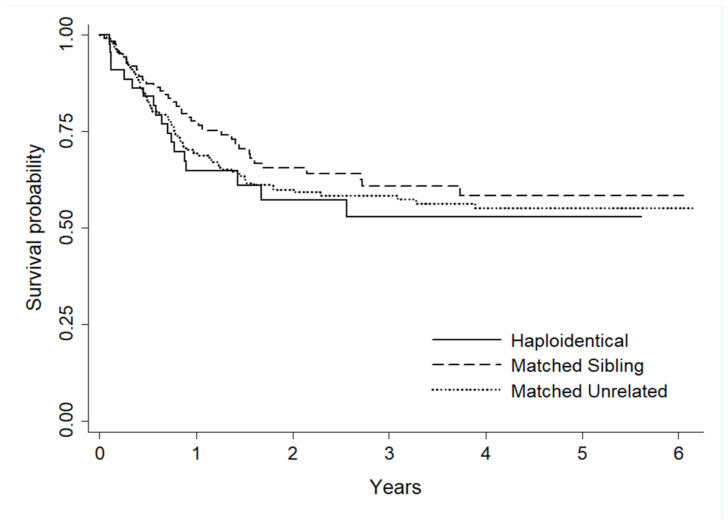
Comparison of survival probability between donor sources.

## Abstract 20 (Poster): Respiratory Viral Illnesses Reduction in Outpatient Stem Cell Transplant Recipients during the COVID-19 Pandemic

**Kalina Abrol ^1^, Carey Landry ^1^ and Chris Bredeson ^1,2^**

^1^ The Ottawa Hospital Cancer Program, Ottawa, ON, Canada
^2^ Department of Medicine, Division of Hematology, The University of Ottawa Ottawa, ON, Canada

**Background:** Since stem cell transplantation became a standard-of-care treatment for blood cancers, infectious complications continue to be the most significant cause of treatment-related mortality within the first 100 days post-transplant. Respiratory Viral Illnesses (RVIs) are particularly concerning due to high communicability during peak seasons and severe patient immune impairment during treatment.
**Purpose:** Robust infection control programs are essential to minimize nosocomial outbreaks. In pre-COVID times, The Ottawa Hospital Transplantation and Cellular Therapy (TCT) Program enforced several protocols to reduce infectious disease transmission. This study assessed whether COVID-19-associated increased infection control measures have decreased overall instances of RVIs in stem cell transplant patients.
**Methods:** Prior to COVID-19, a two-visitor policy was in effect for the inpatient ward, and routine staff hand hygiene audits were performed. In COVID-19 wave one, patients were quarantined on the ward without visitors, and the TCT Day Hospital was shuttered on 17 March 2020, with reopening 26 June 2020. Effective March 2020, inpatients are not permitted to leave their rooms without masks and cannot ambulate off-unit without approval. All staff have been required to always wear masks onsite during the pandemic. Pre-pandemic and pandemic infection data for all patients were collected from the hospital electronic medical record. Pre-pandemic patients were defined as having had a transplant to admission day 100 between 11 March 2019 to 11 March 2020 (143 total), and pandemic period patients were those admitted for transplant from 11 March 2020 to 31 March 2021 (185 total). Outpatients were defined as anyone spending > 1 day in the Day Hospital.
**Results:** RVI instance was significantly reduced among outpatient allogeneic and autologous stem cell transplant recipients during the pandemic period, with a total RVI decrease from 11.4% to 1.5% (*p* = 0.0013). There was no change in RVI instance among patients fully admitted for their transplant up to 100 days (*p* = 0.7504). There was one COVID-19 outbreak on the in-patient ward during the pandemic, resulting in 4 patient cases and 2 patient deaths.
**Conclusions:** COVID-19 inspired practices will provide the ongoing benefit of decreased RVI in the TCT Day Hospital Program. As inpatient infection control procedures were already rigorous pre-pandemic, overall RVI occurrences did not significantly increase during the pandemic period. The observed reduction in out-patient RVIs is likely attributed to a combination of factors, including increased infection prevention/awareness practices, adherence to community lockdown measures, and an overall decrease of non-COVID-19 community RVIs. Continued patient infection prevention education is essential. These data support the continued presence of a single visitor on the ward when stringent infection control measures are followed.

## Abstract 21 (Poster): Paraparesis and Petechiae Post Allogeneic Bone Marrow Transplant for Severe Aplastic Anemia: A Case Report

**Robert McDougall ^1^, Ayman Sayyed ^1^, Zoe Evans ^1^, Armin Gerbitz ^1^, Jason Lazarou ^2^, Jonas Mattsson ^1^ and Rajat Kumar ^1^**

^1^ Hans Messner Allogeneic Blood and Marrow Transplantation Program, Princess Margaret Cancer Center, Toronto, ON, Canada
^2^ Neurology, Mount Sinai Hospital, University of Toronto, Toronto, ON, Canada

In allogeneic bone marrow transplantation (BMT), neurological complications are uncommon, and Guillain-Barre Syndrome (GBS) is rare (Yoshida T et al. eNeurologicalSci 2016; Sakellari I et al. J Neurol 2019). Late onset immune thrombocytopenia is also infrequent. To the best of our knowledge, there is no reported case of simultaneous GBS and immune thrombocytopenia post allogeneic hematopoietic cell transplantation (HCT).
A 28-year-old female with severe aplastic anemia received a 10/10 MUD BMT after failure of immunosuppressive therapy. Post-transplant, she developed acute GVHD which resolved with treatment. Nine months post-transplant she was stable, on prophylactic tacrolimus, with Hb 140, WBC 8.3, neutrophils 5.7 and platelets 230. She received vaccinations for HPV, DTap-IPV-HiB.
Three weeks later, she developed headaches, blurred vision, lower limb muscle weakness, difficulty in walking and a petechial rash. These symptoms worsened over 2–3 weeks. Her neurological exam revealed weakness of hip flexors, hip abductors, and dorsiflexors of the ankle, with power 3–4/5. The deep tendon reflexes at knee were normal, but ankle jerks were diminished. There was no sensory loss. Cranial nerves and upper limbs were normal. She had petechiae on lower limbs and then developed menorrhagia. Differential diagnosis included: PRES, GVHD affecting CNS/peripheral nerves, Leukoencephalopathy, Graft failure. Tacrolimus was stopped.
Investigations—Neurological—MR of brain and spine were normal. There was striking enhancement of the cauda equine nerve roots in the post contrast phase and the nerve roots appeared thickened. CSF revealed high protein 1.62 g/L and normal WBC 2X10E6/l, negative on viral and infection screen. Nerve conduction demonstrated prolonged tibial and peroneal motor distal latencies.
Hematological (lowest counts): Hb 119 g/L, WBC 6.0 × 10^9^/L, neutrophils 4.8 × 10^9^/L and platelets 10 × 10^9^/L, Bone marrow biopsy was normocellular with unremarkable morphology. Chimerism CD3 94.9% donor and CD 98.7% donor
A diagnosis of GBS with Immune Thrombocytopenia post vaccination was made. Treatment—She was treated with IVIG 1 gm/kg for 2 days. Her platelets started rising in 1 day, were 114 in 4 days and reached a peak of 221 in 14 days. There was gradual improvement of the weakness, and the patient could walk unsupported in 3 to 4 days. After two weeks of the IVIG treatment, there was recurrence of lower limb weakness, with inability to stand and walk unsupported. A second course of IVIG was administered with gradual improvement starting within 4 days. Repeated nerve conduction studies showed gradual improvement.
The possible trigger for this clinical presentation was the vaccination.
Aim: To share our experience of this rare complication of simultaneous GBS and Immune thrombocytopenia with a short review of literature. Vaccinations are standard-of-care after HCT but can result in immunological complications. IVIG can lead to rapid improvement.

## Abstract 22 (Poster): Inotuzumab Ozogamicin Post-Transplant for Acute Lymphoblastic Leukemia

**Leland Metheny ^1^, Christina Cho ^2^, Ronald Sobecks ^3^, Navneet Majhail ^3^, Sergio Giralt ^2^, Paolo F. Caimi ^1^, Folashade Otegbeye ^1^, Kirsten M. Boughan ^1^, Brenda W. Cooper ^1^, Molly Gallogly ^1^, Ehsan Malek ^1^, Benjamin Tomlinson ^1^, Aaron Gerds ^3^, Betty Hamilton ^3^, Matt Kalaycio ^3^ and Marcos de Lima ^1^**

^1^ Adult Hematologic Malignancies & Stem Cell Transplant Section, University Hospitals Seidman Cancer Center, Cleveland, OH, United States
^2^ Stem Cell Transplant Program, Memorial Sloan Kettering Cancer Center, New York, NY, United States
^3^ Cleveland Clinic Stem Cell Transplant Program, Cleveland, OH, United States

**Background:** Allogeneic transplant (alloHCT) is a curative therapy for patients with acute lymphocytic leukemia (ALL). However, the curative potential of alloHCT is hampered by relapse, which is the major cause of treatment failure. Risk factors for relapse include minimal residual disease (MRD) before or after allogeneic transplantation, patients transplanted in second complete remission or beyond, and reduced intensity conditioning (RIC) regimens. Overall, relapse rates for these patients are in the range of 30% to 50% with the majority of these relapses occurring within the first year after alloHCT. After relapse, the options for disease control are limited and overall survival rates are poor. Currently, there are no standard post-transplant therapy for patients with ALL to reduce the likelihood of relapse. Post alloHCT maintenance approaches may provide time for the graft-versus-leukemia effect to develop, while possibly treating minimal-residual disease, prolonging leukemia-free-survival and decreasing relapse rates.
Inotuzumab ozogamicin (INO) is a CD22 monoclonal antibody bound to calicheamicin which has been shown to have significant activity against relapsed ALL. INO has also been used in patients with relapsed/refractory ALL in a phase 3 randomized clinical trial with an overall CR rate of 81% in the INO arm compared to 29% in the standard arm. We hypothesize that low dose post-alloHCT maintenance therapy of INO will be safe and will reduce relapse rates after alloHCT for ALL. Once the appropriate dose has been found, the study will expand to a phase II.
**Methods:** Eligibility included patients aged 16–75 who have undergone all-HCT for CD22^+^ ALL in complete remission, have a high risk for relapse after alloHCT, have adequate graft and organ function, are between day 40 and 100 post-alloHCT, do not have active grade III/IV GVHD or any active GVHD of the liver, and have no history of VOD. The primary objective of this phase I clinical trial is to define a recommended phase II dose (RP2D) of INO. Secondary objectives include describing the safety profile of INO post-alloHCT, determining the rate of veno-occlusive disease in this setting, evaluating non-relapse mortality (NRM), disease free survival (DFS) and overall survival (OS) at 1 year post-alloHCT, determining the incidence of myeloid toxicity and secondary graft failure, and determining if INO at these doses are effective at eradicating MRD in this cohort of patients.
The trial design is a 3 + 3 model. The treatment consists of a total of 28-day cycles of INO starting at dose level 0 (0.3 mg/m^2^) given on day 1 for up to 12 cycles The first cycle must be initiated between day 40 and 100 after alloHCT. Dose limiting toxicities (DLT) include VOD, prolonged cytopenia (more than 28 days), and death.
**Results:** We have completed enrollment for the phase I portion of this clinical trial and defined the phase II dose at 0.6 mg/m^2^. 19 patients have been treated. One patient went off treatment after 1 cycle of INO (patient choice, replaced) and five went off treatment after 3 cycles of INO (patient choice and physician choice). Pt 8 experienced a DLT due to prolonged thrombocytopenia. At 1-year post-alloHCT no patients have relapsed and OS is 100%. There were 2 relapses after 1-year post-alloHCT.
**Conclusions:** Low dose INO post-alloHCT maintenance is safe and the RP2D has been determined. The phase II portion of this trial is now open to accrual. The low level of disease recurrence in this cohort of high risk ALL patients is very promising.

## Abstract 23 (Oral): HCT Frailty Scale for Adults Undergoing Allogeneic Hematopoietic Cell Transplantation

**Maria Queralt Salas ^1,2,3^, Eshetu G. Atenafu ^4^, Esrak Al-Shaibani ^1,2^, Ora Bascom ^2^, Leeann Wilson ^2^, Carol Chen ^1,2^, Ivan Pasic ^1,2^, Arjun D. Law ^1,2^, Wilson Lam ^1,2^, Dennis (Dong Hwan) Kim ^1,2^, Armin Gerbitz ^1,2,^ Auro Viswabandya ^1,2^, Jeffrey H. Lipton ^1,2^, Fotios V. Michelis ^1,2^, Jonas Mattsson ^1,2^, Shabbir Alibhai ^5^ and Rajat Kumar ^1,2,^**

^1^ Department of Medicine, Section of Medical Oncology and Hematology, University of Toronto, Toronto, ON, Canada
^2^ Hans Messner Allogeneic Blood and Marrow Transplantation Program, Division of Medical Oncology and Hematology, Princess Margaret Cancer Centre, University Health Network, Toronto, ON, Canada
^3^ Hematopoietic Cell Transplant Unit, Institut Clínic d’Hematologia i Oncologia, Fundación Josep Carreras, Hospital Clínic de Barcelona, Barcelona, Spain
^4^ Department of Biostatistics, Princes Margaret Cancer Centre, University Health Network, Toronto, ON, Canada
^5^ Department of Medicine, University Health Network, Toronto, ON, Canada

**Introduction:** Hematopoietic cell transplantation (HCT) Frailty Scale for overall survival (OS) prediction has been designed by our Institution to identify fit, pre-frail, and frail candidates for alloHCT at the time of the first consultation. This scale consists of measurement of eight variables: Clinical Frailty Score, Instrumental Activities of Daily Living scale, Grip strength score, Timed Up and Go Test, Self-Rated Health questionnaire, a question on Falls, albumin level and C-reactive protein level, in a median time of 5–6 min.
**Statement of the main thesis:** This study reports the results of this scale applied to a cohort of 298 patients undergoing alloHCT between 2018 and 2020 and compares its predictive ability with HCT-CI and KPS.
**Summary:** Frailty was evaluated prospectively at first consultation in all adults referred for alloHCT after providing informed consent.
HCT Frailty Scale application: A normal result obtained from each of the eight variables is scored 0. For any abnormal result, a proportional weight score is given to each respective index variable. This value was defined based on the HR coefficient from the multivariate Cox model for OS estimated including the eight variables of interest. The total score value is calculated as the weighted sum of values of the eight indices evaluated and ranges from 0 to 10.5. Based on the score obtained from the application of the scale, patients are classified in one of the following three groups: Fit: score ≤ 1/Pre-Frail: 1 < score < 5.5/Frail: score ≥ 5.5. After dividing the study cohort in the following groups: frail vs. pre-frail and fit, KPS 70–80% vs. 90–100%, and HCT-CI score > 3 vs. 0–3; the predictive ability of the HCT Frailty Scale was calculated using Harrell’s Concordance Statistics and ROC curves, and compared with the predictive ability of HCT-CI and KPS.
Median age was 58 years (range: 19–76), 53 (17.8%) patients had a KPS between 70–80%; and 54 (19.2%) a HCT-CI > 3. The HCT Frailty Scale classified patients as fit: 103 (34.5%), pre-frail: 148 (49.6%), and frail: 47 (15.7%). The estimated 1-year OS of each group was 86.2% (95% CI 76.8–91.9), 73.8% (95% CI 65–80.7), and 50.9% (35.4–64.4) (*p* < 0.001). The estimated 1-year NRM of each group was 5.4% (95% CI 2–11.5), 15.3% (95% CI 9.7–22.1) and 37.7% (23.6–51.7) (*p* < 0.001).
The predictive ability for OS of the HCT Frailty Scale was found to be higher than the predictive ability of HCT-CI score and KPS (Harrel’s Concordance Index: 60.0% vs. 54.5% and 52.8%, respectively).
**Conclusions:** The HCT Frailty Scale has been specifically designed to be applied in routine clinical practice to assess adult candidates for alloHCT across all ages. This innovative frailty scale is calculated as the weighted sum of values of eight domains and is a valuable predictive tool when evaluated at the first consultation. The use of this scale can identify frail and pre-frail patients prior to alloHCT.

## Abstract 24 (Poster): A Retrospective Review of the First 15 Months of the CAR-T Program from the Princess Margaret Cancer Centre, Canada

**Carmel Waldron ^1^, Andrew Winter ^1^, Anca Prica ^1^, Michael Crump ^1^, John Kuruvilla ^1^, Vishal Kukreti ^1^, Robert Kridel ^1^, Arjun D. Law ^2^, Ivan Pasic ^2^, Sam Saibil ^1^, Ivan Landego ^1^, Wilson Lam ^2^, Rhida Bautista ^2^, Eshrak Al-Shaibani ^2^, Christine Chen ^1^ and Sita Bhella ^1^**

^1^ Division of Medical Oncology and Hematology, Princess Margaret Cancer Centre, Toronto, ON, Canada
^2^ Hans Messner Allogeneic Blood and Marrow Transplant Unit, Princess Margaret Cancer Centre, Toronto, ON, Canada

**Background:** The SCHOLAR-1 study found that patients with refractory diffuse large B-cell lymphoma (DLBCL), transformed follicular lymphoma (tFL) and primary mediastinal B-cell lymphoma (PMBCL) had a median overall survival (OS) of 6.3 months with high-risk subgroups having a median OS time of 3.8 months. CD19-directed Chimeric Antigen Receptor T-cell therapy (CAR-T) harnesses the anti-neoplastic potential of autologous T-lymphocytes by genetically modifying them to target the lymphoma CD19 antigen. Seminal anti-CD19 CAR-T clinical trials achieved overall response rates of 65% (at one year) and complete response rates of up to 54% in cases of chemorefractory lymphoma.
**Purpose:** The primary aim of this study is to assess the OS and Progression Free Survival (PFS) of patients undergoing CAR-T cell therapy at Princess Margaret Cancer Centre (PMCC).
**Methods:** Consecutive patients assessed for CAR-T therapy in the PMCC from the 2 June 2020 to the 1 September 2021 were retrospectively reviewed.
**Results:** 69 patients were referred for CAR-T, of these 53 (76.8%) received CAR-T, of whom 11/53 (20.8%) were from outside of Ontario (ON), 42/53 (79.2%) were from ON (UHN- 33/53 (62.3%); Not UHN 9/53 (16.9%)). 16 patients did not receive CAR-T; Failed CAR-T manufacturing: 3/16, failed lymphocyte collection: 2/16, low performance status: 6/16, CNS disease: 3/16, patient declined: 2/16.
The median age at the time of CAR-T infusion was 61 years (range 23–82). Males 32; Females 21. The indications were; DLBCL: 30/53 (56.6%), tFL: 13/53 (24.6%), PMBCL: 4/53 (7.5%), high-grade B-cell Lymphoma 5/53 (9.4%) and B-cell lymphoma, unclassifiable with features intermediate between DLBCL and cHL 1/53 (1.9%).
A total of 42/53 (79.2%) patients underwent bridging therapy: Steroids alone 6/42 (14.3%), radiation alone 24/42 (57.1%) chemoradiation 6/42 (14.3%) and chemotherapy alone 6/42 (13.3%). Chemotherapy regimens used were: ICE: 5, Ritux-Pola: 1, Pola-BR: 2, Etoposide (oral): 1, GDP: 2 and GemOx: 1. One patient (1.9%) did not receive lymphodepleting chemotherapy, fludarabine/cytarabine was used in 51/52 (98%) and bendamustine was used in one case (1.9%). In total 28/53 (52.8%) received tisagenlecleucel (KYMRIAH) and 25/53 (47.2%) axicabtagene ciloleucel (YESCARTA).
The median CAR-T inpatient stay was 14 days (range 9–67). As of 15 May 2022, 19/53 (35.8%) patients had died with a median time to death of 106 days (range 10–374).
**Conclusions:** CAR-T has revolutionized the treatment of patients with chemo-refractory large B cell lymphoma. This study highlights the successful implementation of a CAR-T program within Canada. This data will help develop and guide further CAR-T programs. Currently we are analyzing the OS and PFS data and will present our findings at the national meeting.

## Abstract 25 (Poster): CD56^bright^ NK Cell and CD21^low^ B Cell Predict Response of cGvHD Therapy: Results of a Multicentre Phase II Clinical Trial (The CARE Trial)

**Vaishnavi Parthasarathy ^1^, Sayeh Abdossamadi ^1^, Barnaby Malong ^1^, Elena Ostroumov ^1^, Imran Ahmad ^2^, Denis-Claude Roy ^2^ and Kirk R. Schultz ^1^**

^1^ British Columbia Children’s Hospital Research Institute, University of British Columbia, Vancouver, BC, Canada
^2^ Département de médecine, Université de Montréal, Montreal, QC, Canada

**Background:** Chronic graft versus host disease (cGvHD) is a major cause of non-relapse mortality in hematopoietic stem cell transplantation (HSCT) patients. The CARE Trial (Continuous Alloreactive T Cell Depletion and Regulatory T Cell Expansion for the Treatment of Steroid- Refractory or Dependent Chronic GvHD) was a Multicentre Phase II clinical trial through the CTTC network. The trial evaluated in patients with steroid refractory cGvHD an alternative approach to extracorporeal photopheresis (ECP) with TH9402-based photodynamic therapy (TH-PDT), a new type of phototherapy that does not involve frequent apheresis sponsored by the Canadian Donation and Transplantation Research Program (CDTRP) of which CTTC was a collaborator. A primary mechanism for TH-PDT therapy was hypothesized to work by increasing Tregs in vivo although CD56^Bright^ NK cells, for example, have been shown to be associated with a response in ECP (Iniesta et al., 2018) in effect to ECP. Other immune cellular mechanisms are also well described in cGvHD including populations of naïve and autoimmune B cells, naïve T cells, and CD56^bright^ NK cell populations.
**Purpose:** To evaluate the impact of TH-PDT enrolled on the CARE Trial on non-Treg populations known to be associated with cGvHD.
**Methods:** PBMC were analyzed for patients from the trial at four time points post ECP treatment W0, W4, W14, W19. Multi-parametric Flow cytometry analyses were performed for several immune markers CD19, CD21, CD38, CD24, IgD, CD27, CD5, CD10 and CD3, CD16, CD56, CD335, CXCR3, CD8, Perforin, Granzyme B, and Granzyme K on BD LSR Fortessa X20 and analyzed by Kaluza 2.1. Statistical Analysis was performed using GraphPad Prism (Mann–Whitney Test). We evaluated the following populations CD19^+^ B cell, CD21^low^ CD19^+^ B cells, and CD27^+^CD10^+^ CD21^low^ B cells, Transitional B cells, CD56^bright^ NK cells, CD56^dim^ NK cells, CD3^+^ T cells, and CD3^+^CD8^+^ cytotoxic T cells populations known to be involved in GvHD.
**Results:** Although statistical significance could not be drawn due to the low sample size, we saw trends in two immune cell populations. We observed that increased levels of CD21^low^ B cells at baseline correlated with a response (Mean = 29.89% of CD19^+^ B cells; *n* = 10) compared to the non-responders (Mean = 5.59% of CD19^+^ B cells; *n* = 3; *p* = 0.1608). We also found elevated levels of CD56^bright^ at baseline associated with any response (Mean = 18.66% of total NK cells) compared to non-responders (Mean = 10.35% of total NK cells; *p* = 0.1608). We also predict that increased ratio of CD56^bright^ to CD56^dim^ (*p* = 0.1608) tends to facilitate response to this therapy.
**Conclusions:** These findings require confirmation in larger cohorts, but we show that the presence of high levels of CD21^low^ B cells appeared to predict rapid response and elevated CD56^bright^ NK cells at W0 appear to correlate with any type of response to therapy in this 13-patient study.

## Abstract 26 (Poster): Demand and Usage of Unrelated Donor Products for Allogeneic Hematopoietic Cell Transplantation during the COVID-19 Pandemic: A Canadian Blood Services Stem Cell Registry Analysis (Award Recipient—Clinical Trials/Observations)

**David S. Allan ^1,2,3^, Meagan Green ^1^, Gail Morris ^1^, Jason Weiss ^1^, Nicholas Dibdin ^1^, Dena Mercer ^1^ and Matthew D. Seftel ^1,4^**

^1^ Stem Cells, Canadian Blood Services, Ottawa, ON, Canada
^2^ Blood and Marrow Transplantation, Hematology, Department of Medicine, The Ottawa Hospital, Ottawa, ON, Canada
^3^ Department of Medicine and Biochemistry, Microbiology & Immunology, Faculty of Medicine, University of Ottawa, ON, Canada
^4^ Department of Medicine, Faculty of Medicine, University of British Columbia, British Columbia, BC, Canada

**Background and Objectives**: Understanding changes in demand and usage of unrelated allogeneic HCT donors during the COVID-19 pandemic is needed to optimize pandemic preparedness of registry and donor collection services. The aim of this study was to understand the extent to which the pandemic has impacted demand and usage of unrelated donors and cord blood units at Canadian Blood Services.
**Methods**: Data regarding stem cell donor interest and product usage for unrelated allogeneic hematopoietic cell transplantation was retrieved from the database at Canadian Blood Services using de-identified anonymous information.
**Results:** Unrelated donor searches for Canadian patients remained unchanged by the pandemic, reflecting stable demand. The number of unrelated allogeneic transplants performed within Canada also remained stable while the number of cord blood transplants increased, chiefly for pediatric patients. Requests for donor verification typing, a first signal of potential interest, increased from domestic centres during the first 6 months of the pandemic and decreased from international centres, before returning to baseline levels. The proportion of transplants for Canadian patients that used stem cell products procured from Canadian donors increased between 3–6 months after the start of the pandemic before returning to baseline and appears to be increasing again more than 1 year after the start of the pandemic. Use of cord blood units for Canadian pediatric patients increased and remains elevated.
**Conclusions:** Demand for unrelated adult HCT donors has remained stable despite the evolving pandemic with a transient and recurring increased interest and usage of domestic adult donors. Use of cord blood units for pediatric patients has increased and remains elevated. Registries and donor collection centres should maintain capacity to expand services for domestic donor collection during pandemics to offset threats to international donor usage.

## Abstract 27 (Poster): Manufacturing Commercial Axicabtagene Ciloleucel for Canadian Patients: A Retrospective Analysis

**Sue Z. J. Li ^1^, Didier Hallard ^2^, Marie Pierre Fontaine ^1^, Iwona Grzegorczyk-Manuel ^1^ and Lieven Billen ^1^**

^1^ Kite, a Gilead Company, Mississauga, ON, Canada
^2^ Kite, a Gilead Company, Hoofddorp, The Netherlands

**Background:** Axicabtagene ciloleucel (axi-cel), an autologous anti-CD19 chimeric antigen receptor T-cell therapy was approved by Health Canada on 13 February 2019 for use in adults with relapsed or refractory large B-cell lymphoma (LBCL) after ≥2 lines of systemic therapy, including diffuse large B-cell lymphoma (DLBCL) not otherwise specified, primary mediastinal large B-cell lymphoma (PMBCL), high grade B-cell lymphoma, and DLBCL arising from follicular lymphoma. We previously reported the experience in manufacturing and supplying commercial axi-cel lots in European countries and Israel (van del Wiel et al., EBMT 2021 P012).
**Purpose:** To describe the experience in manufacturing and supplying commercial axi-cel lots to patients treated in Canada.
**Methods:** Fresh apheresis materials were collected from patients intended to receive commercial axi-cel at authorized treatment centres (ATCs) in Canada and shipped to El Segundo, CA, United States for manufacturing. If additional apheresis was needed for remanufacturing, the first apheresis date was considered for each patient, and is subsequently referred to as that patient’s lot. The finished product lot remained at the manufacturing facility until Quality Assurance (QA) release as per Health Canada’s Good Manufacturing Practice requirements and was then shipped to the ATC, under direct QA import monitoring. This analysis includes 2 years of experience from the date of the first patient who was apheresed to receive commercial axi-cel.
**Results:** From first apheresis after marketing authorization, 7 November 2019, until 5 November 2021, 92 patients were registered on the Kite Konnect^®^ website and provided apheresis material for axi-cel manufacturing. The median time (Q1, Q3) from apheresis to QA release was 18 days (17, 18), with a range of 17–57 days. The median time (Q1, Q3) from apheresis to finished product delivery was 21 days (21, 23), with a range of 19–60 days.
Among the 92 patients who underwent apheresis, 91 axi-cel lots (99%) were released, which included 90 commercial lots (99%) released by QA and 1 lot (1.1%) that was out of specification (OOS). The OOS lot was released based on a risk/benefit assessment according to Health Canada’s Biologics Lot Release Program. One lot (1.1%) was cancelled by the ATC prior to release. Among released lots, 90 lots (99%) were delivered to ATCs, with one lot (1.1%) cancelled by the ATC after QA release but prior to shipment.
Remanufacturing was required either from excess frozen PBMCs for 1 lot (1.1%) or from a new apheresis for 1 lot (1.1%), the latter representing the longest apheresis to delivery time of 60 days. In both cases remanufacturing yielded product that met product specifications.
  **Conclusions:** The early experience in manufacturing commercial axi-cel for patients treated in Canada shows high manufacturing success, with a reliable time from apheresis to product delivery (median, 21 days) and a very low percentage (1.1%) of out of specification lots.

## Abstract 28 (Poster): A Quality Improvement Initiative of Increasing Capacity for Autologous Stem Cell Transplants

**Uday Deotare ^1,2,3^, Adrienne Fulford ^1,2^, Anargyros Xenocostas ^1,2,3^, Susan Nugent ^1,2^, Susan Reiger ^1,2^, Mark Mussio ^1^, Deanna Caldwell ^1,2^, Cadance Halley ^1^ and Alan Gob ^1,3^**

^1^ London Health Sciences Centre, London, ON, Canada
^2^ Blood and Marrow Transplant Program, London Health Sciences Centre, London, ON, Canada
^3^ Division of Hematology, Department of Medicine, Schulich School of Medicine and Dentistry, University of Western Ontario, London, ON, Canada

**Introduction:** Autologous Stem Cell Transplant (ASCT) patients have an inpatient length of stay (LOS) of 21–28 days. In a resource constrained environment, this leads to increasing wait times and limited ability to do transplants. We tried to create additional capacity for transplants at our institution by studying the root causes of this problem and designing improvements in our own resource constrained environment.
**Hypothesis & Rationale:** Nationwide, there is an increased number of eligible patients for ASCT but with limited available inpatient beds. We used the model for improvement principle in an effort to increase the capacity for ASCT. This consisted of identification of major root causes and selecting a group of interventions focused on the underlying root causes. One of the major root causes evident was increased length of stay (LOS) of ASCT patients. It was planned to address this issue by a series of change ideas implemented by developing and conducting small tests of change called PDSA cycles.
The root causes of increased LOS identified by admission census and serial audits, were then plotted on a Pareto Chart.
**Objectives:** Our main objective or Big Dot aim was to increase the capacity of ASCT at LHSC by 20% by December 2020.
**Methods:** A major root cause of increased LOS of ASCT patients was identified and was targeted for improvement.
The first change idea was to change inpatient Conditioning chemotherapy to outpatient. This was initially carried out for BEAM chemotherapy followed by Melphalan Conditioning. This was successfully carried out by various PDSA ramps consisting of conversion of IP PowerPlan to OP, creation of a modified CoSTARS assessment tool used to determine the risk pathway for readmissions. The subsequent PDSA ramps consisted of education of Incharge Nurses, trainees and patients.
Subsequently early discharge on Day + 15 was undertaken for ASCT patients if the patients were clinically stable. The data of LOS was plotted on a Shewart Process Control Chart “I Chart” using QI Macros software.
**Results:** The median LOS at the baseline was 24.5 days (range: 16–42). After the implementation of the change idea on 10 December 2019 till 5 February 2020, the LOS for ASCT decreased to 16.3 days (range: 11–21). Further, decrease in inpatient stay was achieved with outpatient Melphalan. Subsequently, a multidisciplinary plan was instituted to discharge patients on Day + 15. With all the above three measures, the capacity for ASCT in our institution increased by 22.5% during the study period.
**Conclusions:** Increase in the capacity for ASCT by decreasing the LOS by outpatient conditioning chemotherapy and Early discharge on Day + 15, is possible and feasible in the QI framework. This is an ongoing project at the time of abstract submission and the full outcome of this study would be presented in detail at the CTTC conference.

## Abstract 29 (Oral): Quality Improvement Initiative to Improve Re-Vaccination Rates after Autologous Stem Cell Transplantation (Award Recipient—Laboratory/Quality)

**Corina DeKraker ^1^, Austin Kemp ^1^, Allana Simon ^1^, Cristian Rey ^1^, Henry Chang ^1^, Zara Kiani ^1^, Adrienne Fulford ^2^, Sue Nugent ^2^, Denise Singh ^2^ and Uday Deotare ^1,2,3^**

^1^ Schulich School of Medicine and Dentistry, Western University, London, ON, Canada
^2^ Blood and Marrow Transplant Program, London Health Sciences Centre, London, ON, Canada
^3^ The Centre for Quality, Innovation and Safety, Department of Medicine, Western University, London, ON, Canada

**Background:** Autologous hematopoietic stem cell transplant (ASCT) patients undergo a reset of their immune system, resulting in loss of immunological memory. As such, guidelines recommend they be re-immunized against vaccine-preventable diseases beginning 4–6 months post-ASCT. London Health Sciences Centre (LHSC) performs ~80–90 ASCTs annually for hematological malignancies including multiple myeloma and relapsed lymphomas.
**Objectives:** To increase post-ASCT vaccine compliance at LHSC by 15%, from 72% to 87%, by 30 April 2021.
**Methods:** The background data on post-ASCT vaccine compliance were collected via phone survey and estimated to be 72%. These data were based on 53 patients who had undergone ASCT within the previous 7–27 months, surveyed in December 2020. The root causes of incomplete compliance were detailed (See Figure 1: Ishikawa Fish Bone diagram) and the major causes attributed by patients to problems accessing health care providers and the financial burden of certain vaccines were plotted on a Pareto Chart (Figure 2). We implemented two complementary change ideas via PDSA’s by mailing personalized packets containing information on how patients can access and fund vaccinations.
**Results:** Among the first subgroup of LHSC patients to receive this intervention, vaccine compliance was 95% (*N* = 20 patients) (Figure 3). These patients had undergone ASCT within the previous 7–12 months and were surveyed in April 2021. There were negligible costs involved, and the majority of patients described the intervention as accessible and helpful (Descriptive patient surveys).
**Conclusions & Implications:** Mailing personalized information packets to ASCT patients is a cost-effective and well-received measure that may increase post-ASCT vaccination compliance. Future iterations of this QI study could focus on implementing this measure at other hospital sites and collect data in additional patient subgroups to reinforce the validity of the results from this study.

**Figure 1:**
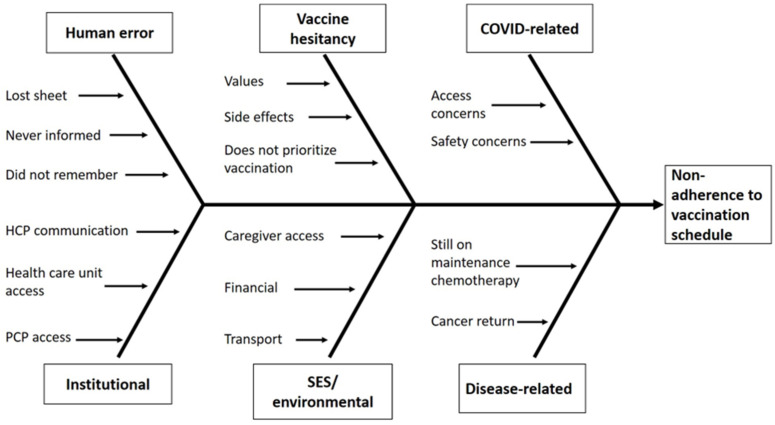
Ishikawa Fish Bone diagram detailing root causes of incomplete compliance.

**Figure 2:**
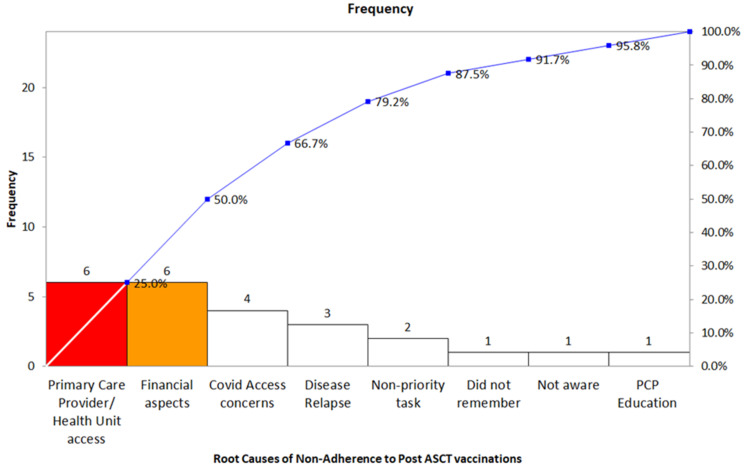
Pareto Chart outlining major causes attributed by patients to problems accessing health care providers and the financial burden of certain vaccines. The most frequent causes are coloured.

**Figure 3:**
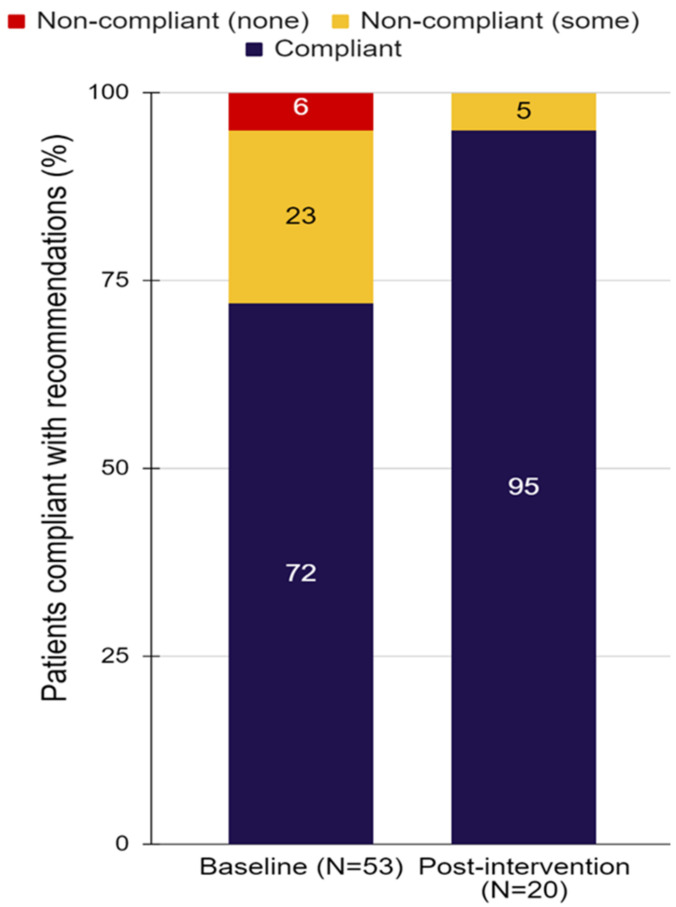
Compliance distribution before and after intervention.

## Abstract 30 (Poster): Development and Utilization of a Hematopoietic Stem Cell Infectious Diseases Risk Matrix (Award Recipient—Laboratory/Quality)

**Steven J. Drews ^1,2^, David S. Allan ^1,3^, Jelena L. Holovati ^1,2^, Tanya Petraszko ^1,4^, Sheila O’Brien ^1^, Kathy Ganz ^1^ and Matthew D. Seftel ^1,4^**

^1^ Canadian Blood Services, Canada
^2^ Department of Laboratory Medicine and Pathology, University of Alberta, Edmonton, AB, Canada
^3^ Department of Medicine, University of Ottawa, Ottawa, ON, Canada
^4^ Department of Medicine, University of British Columbia, Vancouver, BC, Canada

**Background:** Canadian Blood Services (CBS) is responsible for a system of products for transfusion and transplantation that is reliable, safe, accessible, and sustainable. Apart from standard laboratory testing and donor screening, CBS uses additional approaches to ensure the infectious disease-related safety of these products. Hematopoietic stem cell (HSC) products have characteristics that are distinct from transfusable blood components, necessitating a specific safety assessment of these products. To safeguard against not only established pathogens, but also against emerging and re-emerging microbes, CBS developed an infectious disease (ID) matrix.
**Purpose:** To describe the development of a novel Canadian-specific HSC ID matrix at CBS.
**Methods:** In 2019, we undertook a systematic review of new information on ID risks to the HSC supply. We reviewed peer-reviewed publications; infectious disease surveillance online reports; non-peer reviewed scientific information; news media; information from scientific meetings and teleconferences; and person-to-person discussions with peers. Scanning activities included assessing the risk to blood components, source plasma and HSC products. Further refinement occurred after the engagement of peers, subject matter experts and opinion leaders.
**Results:** The ID matrix includes an extensive list of potential pathogens including Babesia, California Serogroup viruses, Chikungunya, Dengue, Ebola, Hepatitis E, Malaria, Avian influenza, SARS-CoV-2, variant Creutzfeldt–Jakob disease, Zika, and Yellow Fever. The matrix details various factors relevant to HSC safety including: Epidemiology; risk of infection associated with exposure; effects of cryopreservation and storage; evidence of donor infection and transmissibility; clinical consequences of transmission; risk mitigation options; impact of mitigation measures on supply; and actions other suppliers have taken or are considering (Figure 1).
**Conclusions:** The ID matrix is a living document that allows users to gauge the significance of established and emerging/re-emerging pathogens throughout the process of donor selection, collection, processing, and transplantation. Constant review of this document will allow for real-time addition of new pathogens. Next steps are to evaluate the impact of the ID matrix by surveying internal and external stakeholders about its utility in the regulatory and clinical settings.

**Figure 1:**
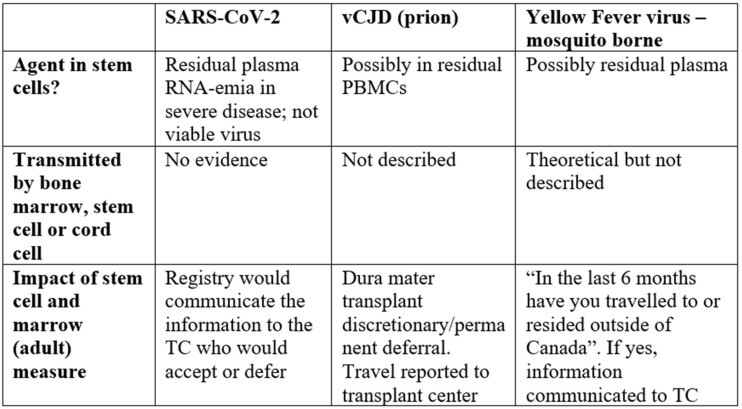
Examples of Matrix Variables and Pathogens. vCJD, Variant Creutzfeldt–Jakob Disease; PBMCs, Peripheral Blood Mononuclear Cells; TC, Transplant Centre.

## Abstract 31 (Poster): Extending Refrigerated Storage of Autologous Peripheral Progenitor Stem Cells Prior to Cryopreservation: One Year Effectiveness Outcomes

**Jelena L. Holovati ^1,2^, Brenda Letcher ^1^, Kelly Murphy ^1^, Nicholas Dibdin ^1^, Kathy Ganz ^1^, Matthew D. Seftel ^1,3^ and Tanya Petraszko ^1,3^**

^1^ Stem Cells, Canadian Blood Services, Canada
^2^ Department of Laboratory Medicine and Pathology, University of Alberta, Edmonton, AB, Canada
^3^ Department of Medicine, University of British Columbia, Vancouver, BC, Canada

**Background:** Hematopoietic Progenitor Cells (HPCs) should be cryopreserved as close as possible to the time point of collection to minimize loss of cell potency and functionality. However, extending the HPCs storage prior to cryopreservation provides many logistical benefits to both apheresis and laboratory operations. Our previous study reported that in vitro quality parameters of cryopreserved HPCs upon 24 h post-collection refrigerated storage met predetermined HPC quantity, viability and potency acceptance criteria, resulting in routine HPC storage for up to 24 h at 2–8 °C prior to cryopreservation at the Canadian Blood Services (CBS) Edmonton stem cell manufacturing facility.
**Purpose:** The aim of this study was to examine both clinical and laboratory effectiveness outcomes one year post-implementation of extended refrigerated storage of autologous HPCs prior to cryopreservation.
**Methods:** Retrospective data analysis included statistical comparison of several quality indicators between 122 and 119 HPC collections processed by our lab one year prior to and post refrigerated storage extension prior to cryopreservation, respectively. Clinical outcomes were also compared for 78 and 83 autologous transplants with HPC products manufactured in our facility in that same time period.
**Results:** There was no statistically significant difference in mean days (d) to ANC engraftment (12.0 ± 1.38 d vs. 12.2 ± 1.18 d, respectively, *p* = 0.3751, *t*-test), nor in the mean days to platelet engraftment (19.3 ± 6.78 d vs. 18.9 ± 5.22 d, respectively, *p* = 0.6472, *t*-test) between one year pre-implementation and one year post-implementation transplant patient cohorts. Incidence of positive sterility rates (% of collection) remained unchanged (*p* = 0.3222, Chi-square test). Similarly, there was no statistically significant difference in incidence of adverse HPC infusion reactions (% of transplants) in the two transplant patient cohorts (2.5 vs. 3.5 %, respectively, *p* = 0.68916, Chi-square).
**Conclusions:** Based upon this post-implementation effectiveness analysis, we demonstrate no negative effects of overnight refrigerated HPC storage on product quality, safety, and transplant clinical outcomes, supporting the recommendation of continued use of 24 h post-collection HPC refrigerated storage in a stem cell manufacturing laboratory compliant with good manufacturing practice regulations.

## Abstract 32 (Poster): Measles Mumps Rubella (MMR) Reactivity and Vaccination Eligibility in Autologous Hematopoietic Stem Cell Transplant (HSCT) Recipients

**Gopika Punchhi ^1^, Aman Sehmbi ^1^, Sharon Pritchard ^1^, Hammad Saif ^1^, Olivia Owen ^1^, Kayla Negus ^1^ and Caroline Hamm ^1,2^**

^1^ Schulich School of Medicine and Dentistry, University of Western Ontario, London, ON, Canada
^2^ Windsor Regional Cancer Center, Windsor, ON, Canada

**Background:** Patients who received a hematopoietic stem cell transplant (HSCT) are at increased risk of infection post-transplant due to their immunocompromised state. Current Canadian guidelines advise that live vaccines should be administered after at least two years post-HSCT, yet many HSCT recipients are not eligible for re-vaccination due to medication contraindications at this time.
**Purpose:** We aimed to assess Measles Mumps and Rubella (MMR) reactivity in autologous HSCT recipients and eligibility for live vaccine administration at two years post-HSCT in those who required MMR revaccination to inform current guidelines on post-HSCT vaccination.
**Methods:** We completed a retrospective chart review of 69 autologous HSCT recipients at the Windsor Regional Cancer Center transplanted from June 2016 to January 2020 to assess post-transplant MMR reactivity. In those without reactivity or with indeterminate status, we assessed eligibility for revaccination based on administration of contraindicated medications at two years post-HSCT, as stated in the most recent Canadian Immunization Guidelines.
**Results:** The most common indication for autologous HSCT was multiple myeloma, with 68% of patients having this diagnosis. Of those with post-HSCT MMR reactivity assessments (*n* = 58), 55%, 72%, and 48% had non-reactive or indeterminate status to measles, mumps, or rubella, respectively. While 67% were reactive to at least one of the three, only 10% of these patients were fully reactive to MMR. Of living patients who required revaccination at 2 years post-HSCT (*n* = 47), 47% were on a contraindicated medication with the most common medications being dexamethasone or prednisone (23%), bortezomib (8.5%) and carfilzomib (8.5%).
**Conclusions:** The majority of autologous HSCT recipients lack MMR reactivity post-HSCT and require revaccination, however many recipients are not eligible for revaccination based on current guidelines. The safety and efficacy of MMR re-vaccination with contraindicated medications should be evaluated to enable a greater proportion of susceptible patients to be re-vaccinated.

## Abstract 33 (Poster): Systematic Scoping Review of Studies Reporting Unexpected Donor-Derived Abnormalities from Recipients of Allogeneic Hematopoietic Cell Transplantation: A Proposed Framework for Donor Disclosure

**Jasmine Candeliere ^1,2^, Aidan M. Kirkham ^2,3^, Risa Shorr ^4^, Gail Morris ^1^, Philip Berardi ^2^, Matthew D. Seftel ^1,5^ and David S. Allan ^1,2,3,6^**

^1^ Stem Cells, Canadian Blood Services, Ottawa, ON, Canada
^2^ Faculty of Medicine, University of Ottawa, Ottawa, ON, Canada
^3^ Clinical Epidemiology Program, Ottawa Hospital Research Institute, Ottawa, ON, Canada
^4^ Medical Library and Learning Services, The Ottawa Hospital, Ottawa, ON, Canada
^5^ Department of Medicine, University of British Columbia, Vancouver, BC, Canada
^6^ Department of Medicine, The Ottawa Hospital, Ottawa, ON, Canada

**Background**: Allogeneic hematopoietic cell transplantation (HCT) is used increasingly to treat blood and immune-based disorders. Post-transplant testing of HCT recipients can lead to unexpected molecular, cytogenetic and other information in donor-derived cells, evoking questions regarding the potential impact on donor health.
**Objective:** To identify the breadth of donor-derived abnormalities identified by testing HCT recipients and the extent to which disclosure and donor follow up are described.
**Methods:** A systematic search and scoping review were conducted following Preferred Reporting Items for Systematic Reviews and Meta-Analyses extension for scoping reviews in OVID MEDLINE and EMBASE (1947 to 24 May 2021).
**Results:** We identified 38 studies (63 donor-recipient pairs) addressing non-leukemic abnormalities, to complement existing literature describing donor cell leukemia and donor-derived myelodysplasia. Donors were unrelated adults (*n* = 20), related family members (*n* = 28), cord blood donors (*n* = 6) or were not reported (*n* = 9). Acquired cytogenetic, molecular, and morphologic abnormalities were reported. Donor origin was confirmed by cytogenetic analysis via karyotyping, FISH, STR-PCR, and other techniques. A disease in donor-derived cells was described in 35 recipients (56.5%). Despite the relevance for testing and disclosure to donors, only 22 cases (32%) mentioned donor follow-up, and 5 cases confirmed that the donor developed a disease associated with the identified abnormality. Unrelated donor disclosure was mentioned in 3 of 26 cases (12%), where the findings were reported back to the registry.
**Conclusions:** Incidental abnormalities identified in transplanted donor cells may contribute to post-transplant risk of illness in the recipient and may be relevant to donor health. A framework for donor disclosure is proposed that incorporates consideration of analytic validity of the testing, potential significance of the finding, and the extent to which the abnormality is actionable. Adoption of effective processes to safeguard both donor and recipient health outcomes related to this issue is needed.

## Abstract 34 (Poster): Assessing and Preparing Patients for HCT: An Environmental Scan of Psychosocial Care

**Zen Gajtani ^1,2^, Maryam Qureshi ^3^, Andrea Feldstain ^1,2^, Jennifer Pink ^1,2^ and Sara Beattie ^1,2^**

^1^ Department of Psychosocial and Rehabilitation Oncology, Tom Baker Cancer Centre, Calgary, AB, Canada
^2^ Department of Oncology, Cumming School of Medicine, University of Calgary, Calgary, AB, Canada
^3^ Counselling Psychology, Werklund School of Education, University of Calgary, Calgary, AB, Canada

**Background:** Hematopoietic Stem Cell Transplant (HCT) frequently demands intensive care needs from patients and their support networks, involves extensive recovery time, and high psychological distress. HCT programs recognize the need to support psychosocial wellbeing, however evidence-based guidance for pre-HCT psychosocial services is sparse.
**Purpose:** We conducted a qualitative environmental scan of programs across Canada, inquiring how transplant programs prepare and assess psychosocial needs for HCT.
**Methods:** HCT program directors across Canada were contacted and asked to identify their psychosocial team members to be contacted for participation. Team members were provided a list of questions about their psychosocial assessment and preparation process with patients and caregivers, to which they could respond by email or interview by phone. Descriptive qualitative analysis was conducted, using steps outlined by Braun and Clarke (2008).
**Results:** The majority of participants were social workers in hospital settings and responded by email rather than phone. Two qualitative themes arose in our analysis. (a) Components and processes of assessment. Most sites included some form of assessment, however for some it was a brief meeting with a social worker about practical needs and for others it was an in-depth meeting including caregivers and covering topics such as practical and financial needs, psychological needs and history, ways of coping, and supports. All participants conducted assessments in an unstructured manner rather than using standardized assessment tools. (b) Design and components of educational sessions. These ranged from providing written materials, live monthly information sessions, to two days of orientation covering the multidisciplinary transplant team and available services.
**Conclusions:** Significant variation exists in the way programs across the country prepare and assess their patients’ psychosocial pre-transplant needs. This qualitative scan identified several strategies used in diverse ways. Further in-depth research on program outcomes across Canada could help identify which strategies are most successful.

## Abstract 35 (Poster): Abstract was withdrawn. Authors were unable to attend and present

## Abstract 36 (Poster): Development of the Integrated Stem Cell Transplant Activity Repository (iSTAR) Tool at Hamilton Health Sciences

**Linda Pati ^1^, Tho Le ^1^, Julie Bosworth ^1^, Edwin Brindle ^2^, Somer Gilbert ^3^ and Pam Baldwin ^3^**

^1^ Regional Oncology & Complex Haematology Analytics (ROCHA), Hamilton Health Services, ON, Canada
^2^ Cellular Therapy and Transplantation, at Hamilton Health Services, ON, Canada
^3^ Transplant Coordinator Unit, Hamilton Health Services, ON, Canada

To perform stem cell transplants and cellular therapy procedures, Hamilton Health Sciences (HHS) must be Regulated by Health Canada and comply with the international standards identified by the Foundation for the Accreditation of Cellular Therapy (FACT). In September 2018, all HHS Haematology reporting & analytical functions were transitioned to the Regional Oncology & Complex Haematology Analytics (ROCHA) team as data management is critical to the coordination of transplant and cellular therapy. As part of this transition, ROCHA were tasked with the development of a bespoke tool to manage all data collection pertaining to Stem Cell Transplant and Cellular Therapy planning that would meet all quality system requirements. As a result, the integrated Stem (cell) Transplant Activity Repository (iSTAR) was created with the purpose of becoming a “one stop shop” tool for users across the program. iSTAR was designed, built and validated against strict acceptance criteria modeling in collaboration with the Stem Cell Coordinators, Quality Manager and Haematology Physicians at Hamilton Health Sciences. During these collaborative interviews with the CTTC team, we were able to identify required data and metrics to support operational activity.
Currently, iSTAR is being used to capture clinical planning information for Autologous transplant, Allogeneic transplant, CAR-T cell therapy, Acute Leukemia and for donor assessment/clearance for Canadian Blood Services. In addition, a module to track funding costs (i.e., graft costs, courier costs, etc.) has been implemented as well as a tracking module which allows users to track outstanding CIBMTR forms and data quality deficiencies. The final module to be implemented in early 2022 will be the Post-Transplant module which will capture patient outcome information as required by FACT, Cancer Care Ontario and HHS researchers.
Some of the key highlights of the iSTAR tool include:
  - improved patient safety
  - identifies potential lost funding
  - reduces manual work performed by Stem Cell Coordinators
  - tracks invoices for reimbursement
  - collection of bespoke operational and financial reports
  - secure web-based tool
  - all data is stored in a secure DataMart repository for easy data extraction
  - sends notices to Clinicians to book 100 day, 6 month and annual follow up appointments
  - integrated change control process to ensure all changes and version coding is traceable
  - Tiered security level privileges ensuring access to correct information
  - Data integrity audits to validate data components
Although iSTAR has been built to reflect the workflow for transplant coordination at HHS, the tool could be customized for any transplant facility.
Related to CIBMTR, plans are being developed to re-create all CIBMTR forms within iSTAR with the goal of electronically submitting the information and the ability to store the data in the iSTAR DataMart.

## Abstract 37 (Oral): Evaluating the Effectiveness of a Training Program to Support Nurses to Administer Cryopreserved Hematopoietic Stem Cells by Intravenous Push Method (Award Recipient—Pharmacy, Nursing, Other Transplant Support)

**Cheryl Page and Jessica Rebeiro**

Hamilton Health Sciences, Hamilton, ON, Canada

**Background:** There are two main methods of infusion for dimethyl sulfoxide (DMSO) cryopreserved hematopoietic stem cell (HSC) products, gravity drip and intravenous (IV) push. DMSO can cause hypersensitivity reactions. Prolonged exposure to DMSO once the cells are thawed increases the risk of cellular damage. Administration of HSCs by gravity drip is slower, causing less DMSO reactions. The faster IV push method reduces cell damage and decreases staff time. An environmental review found that at most centers, nurses administer by gravity drip, and when IV push is required, HSCs are administered by physicians. Our center’s method was IV push by a physician or nurse practitioner (NP). As transplant numbers grew, capacity to perform this skill needed to expand. To maintain the current benefits of the IV push method, registered nurses (RN) were trained to perform this skill.
**Purpose:** To increase capacity at a hematopoietic transplant program, the role of infusing stem cells by IV push method was transitioned from the NPs and physicians to RNs. A successful training program, utilizing simulation, to support these oncology nurses learning this new skill was developed and evaluated.
**Methods:** Nurses attended a four-hour training session, including a didactic portion, simulated infusion, and case studies. Support included a policy, procedure guide, and reaction management guide. At least three infusions were completed with a competency record, precepted by a transplant physician or NP. Evaluation of the training program, performed pre-training, post-training, and follow-up post independent skill performance, utilized the first three levels of the Kirkpatrick Model. The RNs completed evaluations noting patient response.
**Results:** Nurses rated the orientation program positively (Kirkpatrick evaluation level 1). Nurses demonstrated a significant increase in knowledge in cryopreserved HSC infusions from pre-evaluation to post-evaluation (Kirkpatrick evaluation level 2). There was a significant shift in behaviour post-orientation as demonstrated by an increase in three key areas: administration, management of adverse events and management of DMSO reactions (Kirkpatrick evaluation level 3).
**Conclusions:** The role for infusing cryopreserved HSC was successfully transitioned to the RNs, allowing for increased staff capacity, thus supporting the expansion of the transplant program. Evaluation of the training program ensures that learners new to a role translate knowledge into practice.

## Abstract 38 (Poster): Virtual Group Education Pilot for Allogeneic Stem Cell Transplant Candidates

**Carey Landry ^1^, Debbie Bastien ^1^, Sheryl McDiarmid ^1^ and Chris Bredeson ^1,2^**

^1^ The Ottawa Hospital Cancer Program, The Ottawa Hospital, ON, Canada
^2^ Department of Medicine, Division of Hematology, The University of Ottawa, Ottawa, ON, Canada

**Background:** Stem cell transplantation necessitates a comprehensive teaching strategy to ensure the patient understands the procedure, risks, medications, symptom management, and recovery associated with their transplant. Group sessions held outside the clinic environment can enhance the efficacy of the transplant planning appointment while reducing resource needs associated with 1:1 teaching sessions.
**Purpose:** The widespread use of online meetings due to the COVID-19 pandemic afforded a paradigm shift toward remote patient care. A virtual teaching format was trialed for feasibility starting in September 2020 to complement the program’s printed educational material and other education provided by the program clinicians and allied health team.
**Methods:** In-person sessions presented a challenge as 51.5% of allogeneic transplant candidates last year resided >100 km from TOH. Furthermore, COVID-19 restrictions made in-person congregation of immunocompromised patients unadvisable. Thus, a Plan-Do-Study-Act model was applied to launch a virtual group allogeneic (donor) stem cell transplant teaching initiative and facilitate continuous quality improvement. The hospital-acquired enterprise version of Zoom was used, with link generated via the patient electronic medical record (EPIC). An online anonymous survey was developed to assess patient satisfaction and collect feedback. Sessions were held approximately once every two weeks for pre-transplant patients. Consent was obtained and documented in EPIC. The project team met at regular intervals to analyze session feedback and modify the presentation as needed. A standard operating procedure was developed to ensure standardization of educator training and process flow. A French language presentation version was created for enhanced accessibility and made available via YouTube (www.youtube.com/watch?v=7BOdBpl2i4U) on 14 January 2021.
**Results:** 98/105 transplant recipients attended the session between 22 September 2020 to 28 September 2021 (93.3%). Thirty-four attendees (34.7%) completed the survey, with 97.1% reporting they would recommend it to other patients. 91.2% of respondents gave a content quality score ≥ 4/5, with an average technology rating of 4.3/5. Approximately 75 nursing hours were saved in the first year of the program.
**Conclusions:** These sessions offered significant organizational savings in nursing hours, and patients reported feeling better prepared for their treatment afterward, which suggests improved engagement. The program will adopt this education model permanently. Further investigation will assess session impact on patient-reported reflective preparedness for transplant post-discharge and impact on clinic efficiency.

## Abstract 39 (Poster): Donor Advocacy—The Need for an Ethically, Legally, and Morally Informed Framework for Adult Allogeneic Hematopoietic Stem Cell Transplant Donors (Award Recipient—Pharmacy, Nursing, Other Transplant Support)

**Michele Heffering-Cardwell ^1^, Lisa Tinker ^1^, Carole Garmaise ^2^, Jennifer A. H. Bell ^3,4,5,6^, Susan Clarke ^1^, Rajat Kumar ^1,7^ and Jonas Mattsson ^1,7^**

^1^ Hans Messner Allogeneic Transplant Program, Division of Medical Oncology and Hematology, Princess Margaret Cancer Centre, University Health Network, Toronto, ON, Canada
^2^ UHN Health Law and Privacy, University Health Network, Toronto, ON, Canada
^3^ Department of Clinical and Organizational Ethics, University Health Network, Toronto, ON, Canada
^4^ Department of Supportive Care Research, Princess Margaret Cancer Centre, University Health Network, Toronto, ON, Canada
^5^ The Institute for Education Research, University Health Network, Toronto, ON, Canada
^6^ Department of Psychiatry, Dalla Lana School of Public Health, Joint Centre for Bioethics, University of Toronto, Toronto, ON, Canada
^7^ Department of Medicine, University of Toronto, Toronto, ON, Canada

**Background:** Allogeneic hematopoietic stem cell transplant (HSCT) requires the identification of a donor prior to proceeding with transplant. However, in the adult HSCT donor setting, minimal clinical guidance exists to support donors who are incapable of interpreting information and provide informed consent for donation. Consequently, the clinical team is challenged ethically in protecting the rights of the donor while striving for the best therapeutic outcome of the recipient. This scenario was experienced at our facility when it was identified that the HLA-matched sibling did not have the capacity to understand or provide consent to donation. Previous communication with the potential donor had been through the legal substitute decision maker (SDM) of the donor. An ethical concern was that the SDM would be unable to give an unbiased opinion.
**Purpose:** To expand donor care standards to include the diverse needs of all potential adult donors, while adhering to ethical principles and legal responsibilities.
**Methods:** To inform the development of a practice standard for accepting cognitively diverse donors, the authors sought guidance from established programs in the pediatric care setting. A case team was assembled with experts from the HSCT program, bioethics, legal, and communication specialists in collaboration with the potential donor and their family. The team consulted with Canadian Medical Protective Association (CMPA) to obtain legal guidance.
**Findings:** There is a significant gap in donor navigation processes when an adult donor does not have the ability to understand and provide informed consent. Case law on this issue is sparse, and legal opinion appears to vary by jurisdiction, although a similar case [1] outlined that the parents of an adult donor with cognitive disabilities did not have legal authority to consent. Utilizing criteria outlined by The American Academy of Pediatrics Committee on Bioethics, the team adapted a framework to structure guidelines for an incapable adult donor. The defined criteria includes
  (1) there must be no medically equivalent histocompatible relative who is able/willing to donate;
  (2) the donor and recipient must have a strong existing personal and positive relationship;
  (3) there must be a reasonable likelihood the recipient will benefit from HSCT;
  (4) clinical, emotional, and psychological risks to the donor must be minimized;
  (5) ensure minimal risk to the donor,
  (6) donor assent must be obtained as far as possible.
**Outcomes/Recommendations:** The primary recommendation is for a standard framework to ensure equitable and safe care of a diverse donor population that aligns with ethical principles and legal requirements. Further, when it is identified that a donor may not have the mental capacity to provide informed consent, a donor advocate policy should be developed and initiated. Case outcomes are still in process as we are awaiting a decision from CMPA.

**References:**
  1. Clark, R.E. Bone-marrow donation by mentally incapable adults. *Lancet* **1998**, *352*, 1847–1848. https://doi.org/10.1016/S0140-6736(98)04378-5.

